# Chemical-Specific Metabolic Disruption during Early Zebrafish Development is Modulated by the Microbiome

**DOI:** 10.1021/envhealth.6c00215

**Published:** 2026-07-10

**Authors:** Nanna Fyhrquist, Lydia Gugescu, Emma Wincent, Harri Alenius

**Affiliations:** † Institute of Environmental Medicine, 27106 Karolinska Institutet, Stockholm 171 77, Sweden; ‡ Human Microbiome Research Program (HUMI), University of Helsinki, Helsinki 00014, Finland; § Department of Health Sciences, Karlstad University, Karlstad 651 88, Sweden

**Keywords:** metabolomics, environmental toxicology, zebrafish embryos, microbiome–toxicant interactions, germ-free zebrafish, mode of action, multiomics

## Abstract

Environmental contaminants such as per- and polyfluoroalkyl substances, bisphenols, and agricultural fungicides are increasingly recognized as metabolic disruptors, yet how individual chemicals perturb metabolic pathways during early development remains incompletely understood. Here, we used targeted and untargeted metabolomics and pathway analysis in zebrafish embryos to characterize metabolic disruption induced by perfluorooctanesulfonic acid (PFOS), boscalid (BOS), and bisphenol F (BPF). Each compound elicited a distinct metabolic fingerprint, indicating chemical-specific patterns of metabolic stress rather than a uniform response across toxicants. Comparative analysis demonstrated divergence in central metabolic pathways, including mitochondrial metabolism, amino acid and nucleotide turnover, redox balance, and tryptophan metabolism. PFOS induced the most extensive metabolic perturbation, whereas BOS and BPF produced more restricted but distinct metabolic profiles. Comparison of germ-free and conventionally colonized embryos showed that host microbial status modulated the magnitude, but not the direction, of chemical-induced metabolic changes. These metabolic alterations align with exposure-specific transcriptional network modules, supporting distinct chemical modes of action. Together, these findings demonstrate that early life exposure to PFOS, BOS, and BPF results in chemically distinct metabolic stress phenotypes during development and illustrate how integrated metabolomic profiling can resolve compound-specific metabolic effects while accounting for contextual modifiers such as host microbial status.

## Graphical abstract

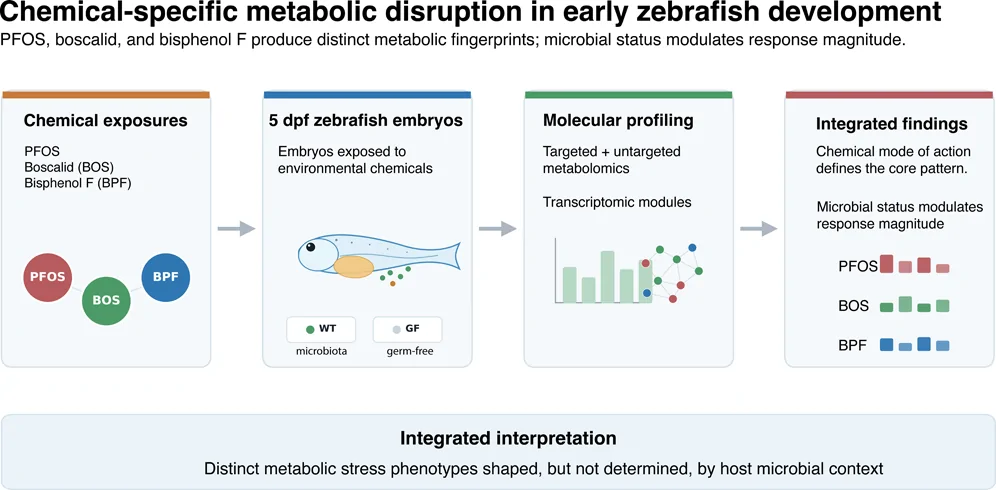

## Introduction

Environmental contaminants such as per- and polyfluoroalkyl substances (PFAS), bisphenols, and certain pesticides and fungicides are increasingly recognized as metabolic disruptors. These compounds have been shown to cause metabolic dysregulation and increased cardiometabolic disease risk in experimental and epidemiological studies.^1–3^ Furthermore, they are frequently detected in the environment, biota, and human populations, and many are associated with altered energy metabolism, mitochondrial dysfunction, endocrine disruption, and developmental toxicity.^4^ Yet, despite the rising concern about these globally prevalent compounds, the mechanistic basis of their toxicity remains incompletely understood.

Early life represents a particularly sensitive window of vulnerability, as developing organisms must coordinate mitochondrial energy production, redox balance, and nutrient utilization to support rapid growth and differentiation.^5^ Disruption of these tightly regulated processes during development may alter metabolic trajectories in ways that predispose individuals to adverse consequences later in life.^6^ Yet it remains unclear to what extent environmental contaminants induce metabolic toxicity and if it is through similar or compound-specific biochemical mechanisms.^3^

Because metabolic perturbations often precede overt developmental or pathological phenotypes, metabolomics offers a powerful approach for resolving the toxicodynamic effects of environmental chemicals during early life. Profiling alterations in amino acid, lipid, nucleotide, redox, and bile acid metabolism provides direct insight into mitochondrial function, nitrogen handling, and systemic energy balance in the developing organism.^7^ However, interpretation of chemical-induced metabolic effects requires consideration of the dynamic and stage-specific metabolic architecture of embryonic development.

During early zebrafish embryogenesis, metabolism is strongly oriented toward rapid cellular proliferation, with high levels of nucleotides and tRNA-related metabolites supporting intense DNA/RNA synthesis and translational activity. As development proceeds, free amino acids progressively accumulate, reflecting increasing protein synthesis and tissue differentiation. By 24 h postfertilization (hpf), rising energetic demand is accompanied by elevated tricarboxylic acid (TCA) cycle intermediates and glucose-related metabolites, indicating increased mitochondrial activity and energy production. Later developmental stages are characterized by the accumulation of metabolic end-products such as creatinine and lactic acid, consistent with maturation of cellular metabolism and waste handling pathways.^8^ Importantly, environmental chemicals can interact selectively with these stage-specific metabolic programs. In humans, PFAS exposure has been associated with altered amino acid metabolism and perturbation of urea cycle pathways, suggesting disruptions in nitrogen balance, mitochondrial function, and cellular energy homeostasis.^9^ These conserved developmental metabolic features make the zebrafish embryo a powerful model for investigating how environmental contaminants perturb metabolic regulation during sensitive windows of development.

The gut microbiome represents an additional factor that can influence host responses to metabolic stress.^10^ The microbiome contributes to nutrient processing, energy harvest,^11^ immune maturation,^12^ and xenobiotic transformation,^13^ and microbial metabolites such as short-chain fatty acids, indoles, and secondary bile acids interact directly with host mitochondrial and redox pathways.^14^ Chemical exposures can in turn alter microbial composition and function, thereby modifying host metabolic responses. Despite this bidirectional interaction, the role of the microbiome as a context-dependent modulator of chemically induced metabolic perturbations during early development remains poorly characterized.

Here, we investigated three mechanistically distinct environmental pollutants—perfluorooctanesulfonic acid (PFOS), bisphenol F (BPF), and the fungicide boscalid (BOS)—to define compound-specific metabolic perturbations during early zebrafish development. Building on the exposure framework of Gugescu et al.,^15^ we applied targeted and untargeted metabolomics to examine whether these chemicals induce distinct biochemical stress programs and exposure-associated transcriptional network signatures. By comparing germ-free and conventionally colonized embryos, we further assessed how the microbiome modulates the magnitude and pattern of chemically induced metabolic disruption. Together, this approach provides mechanistic insight into compound-specific metabolic toxicity and its context-dependent regulation during early development.

## Materials and Methods

### Chemicals

Chemicals used in this study were: PFOS: perfluorooctanesulfonic acid (CAS: 2795-39-3), BPF: bisphenol F (CAS: 620-92-8), BOS: boscalid (CAS: 188425-85-6). All chemicals were of analytical grade quality and were purchased from Sigma-Aldrich (Sweden). DMSO: dimethyl sulfoxide (CAS: 67-68-5) was used as vehicle control.

The three compounds were selected as environmentally relevant and mechanistically distinct exposures with reported developmental and metabolic effects. Using the same exposure paradigm as Gugescu et al.^15^ enabled direct comparison and integration of transcriptomic and metabolomic responses across parallel experiments. PFOS was chosen because it produced robust developmental and transcriptomic effects in the preceding zebrafish study and is a well-characterized legacy PFAS linked to lipid, amino acid, mitochondrial, and bile acid-related metabolism. BPF was selected as a BPA-replacement bisphenol with reported endocrine and neurodevelopmental activity. BOS was included as a succinate dehydrogenase inhibitor fungicide with a mitochondrial respiration-linked mode of action and developmental toxicity in the preceding multiomics screen. Together, these compounds provide mechanistically contrasting contaminants suitable for comparison under identical developmental and microbiome conditions.

### Experimental Design

All nonmetabolomics data were generated in a recently published study.^15^ The experimental conditions and procedures related to these data sets are summarized below; full details are provided in Gugescu et al.^15^

The present study extended this exposure framework by generating targeted and untargeted metabolomics data from the same experimental design and by performing integrative multiomics analyses. Weighted gene coexpression network analysis was conducted using the previously published transcriptomic data, and the resulting modules were integrated with the metabolite profiles generated here. This enabled the identification of compound-specific metabolic fingerprints and coordinated molecular signatures linking chemical exposure, host metabolism, and microbial status.

### Ethics Statement

Zebrafish (*Danio rerio*) embryos were used up to 5 days post fertilization (dpf). At this stage, zebrafish embryos are considered in vitro models and are not protected animals under Directive 2010/63/EU because they have not yet reached independent feeding. Therefore, ethical approval was not required for this study.

### Zebrafish Husbandry and Embryo Exposure

Zebrafish of the AB strain were housed at the Zebrafish Core Facility at Comparative Medicine, Karolinska Institutet. The adult zebrafish were kept in 3.5 L tanks at a density of five fish per liter in a centralized recirculatory aquatic system (# ZB30TK, Tecniplast), kept at 28 °C, with a light/dark cycle of 14 h/10 h. Embryos were produced by mass mating, washed to remove potential debris, and maintained at 28 °C in embryo medium (EM) consisting of 5.0 mmol/L NaCl, 0.17 mmol/L KCl, 0.33 mmol/L CaCl_2_, and 0.33 mmol/L MgSO_4_. The embryos were monitored daily, and dead embryos were continuously removed throughout the exposure period.

Zebrafish embryos were continuously exposed to test chemicals starting at approximately 4 h post fertilization (hpf) in EM at a ratio of 1 mL per embryo. For each condition, 30–50 embryos were pooled per replicate, with five biological replicates per exposure and microbiome status. Exposures were conducted using nominal concentrations of EC_20_ values as described in Gugescu et al.^15^ in either glass Petri dishes or nontreated, sterile, ventilated cell culture flasks (Thermo Scientific, #156800), depending on the experimental end point.

All experimental end points were conducted under identical exposure conditions and developmental stages. Transcriptomic and microbiome data sets were generated from the same exposure experiment using matched individuals collected for different downstream analyses, whereas targeted and untargeted metabolomics was performed in an independent but identically designed experiment using separate embryo pools. Consequently, cross-omics comparisons involving metabolomics were interpreted at the exposure-group level rather than as individual sample-level correlations. Each experiment included its own matched control.

### Generation and Verification of Germ-Free Embryos

Germ-free (GF) zebrafish embryos were generated based on the protocol described by Melancon et al.,^100^ with minor modifications. Briefly, embryos were treated with antibiotic containing EM, including 100 μg/mL ampicillin (#BP1760-25, Thermo Fisher, US), 5 μg/mL kanamycin (#BP906-5, Thermo Fisher, US), and 250 ng/mL amphotericin B (#15290018, Thermo Fisher, US). The embryos were then surface sterilized with 0.1% polyvinylpyrrolidone-iodine solution (PVP-I, Sigma-Aldrich, Sweden) and 0.003% bleach solution under sterile conditions and maintained in sterile EM prior to chemical exposure. Conventionally raised embryos underwent identical handling without sterilization steps to minimize technical confounders. GF status was assessed by aerobic culturing of exposure media in LB broth, with absence of bacterial growth, defined by the OD600 value, used as the inclusion criterion.

### Metabolomics

At 5 days post fertilization (dpf), 50 pooled embryos were collected, rinsed in DPBS to remove residual medium, snap-frozen in liquid nitrogen, and then stored at −80 °C until further preparation. Metabolite extraction was performed using a solvent-based protocol as previously described.^15^ Metabolite profiling was performed by GC–MS and LC–MS analysis at the Swedish Metabolomics Center (SMC), Umeå, Sweden, as described in detail in the Supporting Information Methods.

A targeted, semiquantitative approach was applied to a curated panel of metabolites spanning central carbon metabolism, amino acids and derivatives, nucleotides, organic acids, bile acids, polyamines, lipids (including acylcarnitines, lysophospholipids, and fatty acids), vitamins, and metabolites with known or putative microbial origin or relevance to host–microbiome interactions. The full list of the 251 quantified metabolites is provided in Supporting Information Table S1. Metabolites were putatively identified based on accurate mass, retention time, and comparison with internal and external standards where available.

Raw data processing, log_2_ transformation, median normalization, and initial quality control were performed using the MetaboAnalyst platform.^16,17^ Differential metabolite abundance was assessed using linear modeling with empirical Bayes moderation (limma), with *p*-values adjusted using the Benjamini–Hochberg false discovery rate (FDR). Principal component analysis (PCA) was performed on mean-centered and unit-variance-scaled metabolite abundances. Normalized intensity values were used for downstream analyses comparing WT and GF conditions within each chemical exposure group.

In addition to the targeted analysis, an untargeted metabolomics approach was applied to capture a broader range of metabolic features. Data acquisition was performed using the same GC–MS and LC–MS platforms as described above. Untargeted feature detection, alignment, and deconvolution were conducted using standard workflows at the Swedish Metabolomics Center. Resulting features were filtered based on quality control criteria, log_2_-transformed, and normalized prior to analysis.

Statistical analysis of untargeted data followed the same framework as for targeted metabolites, including linear modeling with empirical Bayes moderation (limma) and Benjamini–Hochberg FDR correction. Multivariate analyses, including PCA, were conducted on mean-centered and unit-variance-scaled data. For visualization, selected features were subjected to row-wise *z*-score scaling for heatmap representation.

### Multivariate and Pathway-Level Analyses

Pathway enrichment analysis was conducted using Ingenuity Pathway Analysis (IPA; Qiagen, Redwood City, CA). IPA was performed separately for all experimental groups, considering both WT and GF conditions. Canonical pathways were filtered based on the adjusted *p*-value <0.05 for the BOS and BPF exposed groups. For PFOS exposed groups, a more stringent cutoff of 0.01 was applied due to the high number of identified pathways. Enriched pathways were subsequently visualized and plotted using the R ggplot2 package (version 3.5.1) and Adobe Illustrator (version 29.3).

### Tryptophan Metabolism Analysis

Metabolites assigned to tryptophan-associated pathways were grouped into kynurenine, serotonin, and indole/AHR-related metabolites. These metabolites were analyzed both as individual abundance values and as tryptophan-normalized ratios to assess relative pathway branching. Heatmaps were generated using row-wise *z*-score scaling to visualize coordinated changes across control and exposure groups. PFOS, BPF, and BOS were included in the same pathway-focused analysis to enable a comparative assessment of compound-specific tryptophan-associated metabolic responses. BOS was included to test whether a succinate dehydrogenase inhibitor with a mitochondrial-respiration-linked mode of action produced tryptophan-associated changes distinct from those induced by PFOS and BPF.

### Weighted Correlation Network Analysis

Weighted correlation network analysis (WGCNA)^18^ was performed on the transcriptomics (RNA-seq) data set produced in Gugescu et al.,^15^ using normalized gene expression profiles to identify transcriptomic gene coexpression modules. Module eigengenes were subsequently correlated with normalized metabolite abundances to assess associations between coordinated transcriptional programs and chemical-induced metabolic alterations. Metabolite abundances were matched to the corresponding exposure group metadata, and trait variables were encoded as numeric or dummy variables as appropriate. The data matrix was organized as samples × genes, and quality control was conducted using the goodSamplesGenes­() function to exclude low-quality samples or genes. Samples were hierarchically clustered using average linkage. Under a signed network model, a soft-thresholding power of 6 was selected using pickSoftThreshold­(), achieving a scale-free topology fit of *R* ^2^ > 0.9. A signed adjacency matrix was constructed using the selected power and transformed into a signed topological overlap matrix (TOM), with 1 – TOM used as the dissimilarity measure for module detection. Modules of coexpressed genes were identified using dynamic tree cutting (cutreeDynamic; minimum module size = 30, deepSplit = 2). Module eigengenes (MEs; defined as the first principal component of each gene module) were calculated and correlated with experimental traits using biweight midcorrelation (bicor), with corresponding *p*-values obtained using corPvalueStudent­(). Correlations between module eigengenes and metabolite abundances were calculated using Pearson correlation coefficients. All analyses were performed in R using the WGCNA package (v1.73) in single-threaded mode (disableWGCNAThreads­(), registerDoSEQ). Outputs included module eigengenes (CSV), module–trait correlation and *p*-value matrices (CSV), a dendrogram displaying gene module assignments, and a heatmap summarizing module–trait relationships (PDF). Because transcriptomic and metabolomic data sets were generated in independent but identically designed exposure experiments, module eigengene–metabolite associations are interpreted at the exposure-group level. Accordingly, the reported relationships reflect concordant exposure responses across conditions rather than covariance between matched individual samples.

### Microbiome-Associated Analyses

At 5dpf, embryos exposed to either DMSO (0.01%) or the modeled EC_20_ value of respective compound of interest were anesthetized using 160 mg/L tricaine, and the guts were dissected using dissection needles as previously described in Gugescu et al.^15^ Briefly, up to five replicate samples per group (each sample consisting of a pool of 10 dissected guts) were collected and the DNA was extracted using the QIAamp DNA Mini kit (#51304, QIAGEN, Germany). To assess microbial community composition, high-resolution full-length 16S rRNA gene sequencing was performed using the PacBio platform (Novogene, UK) across all treatment groups to enable species-level taxonomic resolution.

Pacific Bioscience data were demultiplexed, and we used the DADA2 workflow to process the subsequent circular consensus sequencing (CCS) data.^19^ Briefly, PCR primers and low-quality sequences were discarded; the remaining reads were then denoised, and the chimeras were removed. Taxonomy was assigned using a naïve Bayes classifier trained on the SILVA 16S rRNA gene reference database (release 138) and implemented in QIIME 2.^20,21^ The final ASVs count table was derived from the DADA2 workflow and used for statistical analysis in R (v4.2.1). To identify microbial compositions that characterize different experimental groups, we performed random forest (RF) classification using the R package randomForest (v4.7-1.2) with 5 repeats of 10-fold cross-validation. Heatmaps were generated using the pheatmap package (v1.0.12). Shannon diversity indices and principal coordinate analysis (PCoA) were calculated using the R package Vegan (v2.6.4).

### Statistical Analysis

All statistical analyses were performed using R. Unless otherwise stated, *p*-values were adjusted for multiple testing using the Benjamini–Hochberg false discovery rate (FDR) method, with FDR <0.05 considered statistically significant. Univariate comparisons of metabolite abundances were conducted using appropriate parametric or nonparametric tests depending on data distribution, while multivariate analyses included principal component analysis and correlation-based approaches. Data visualization was conducted using established R packages for multivariate analysis and heatmap generation. Exact statistical tests, thresholds, and sample sizes are specified in the corresponding figure legends.

## Results

### Chemical Exposures Induce Distinct and Structured Metabolic Fingerprints during Early Development

Targeted metabolomic profiling revealed clear and chemical-specific alterations in zebrafish embryos exposed to boscalid (BOS), bisphenol F (BPF), and perfluorooctanesulfonic acid (PFOS) (Figure 1). UpSet analysis identified a core set of 67 metabolites altered by all compounds, while each chemical also produced distinct signatures (PFOS: 21 unique features; BPF: 10; BOS: 9) (Figure 1A). Principal component analysis showed robust separation between exposure groups along the first two principal components, accounting for 36.0% and 19.7% of the total variance, respectively (Figure 1B).

**Figure 1:**
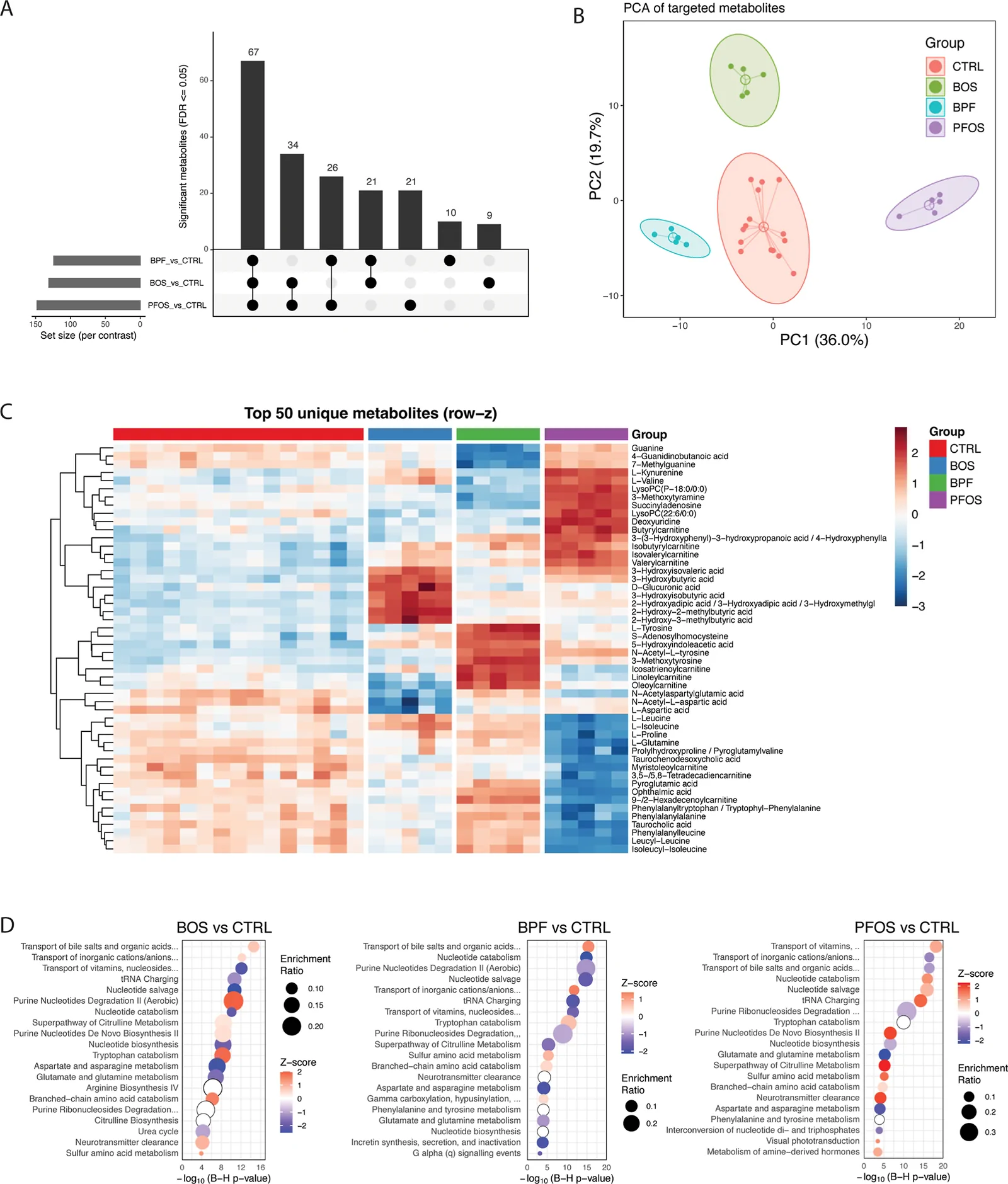
Targeted metabolomics reveals distinct metabolic alterations following exposure to boscalid (BOS), bisphenol F (BPF), and perfluorooctanesulfonic acid (PFOS). (A) UpSet plot showing overlap of significantly altered metabolites (FDR <0.05) in each exposure group compared with control (CTRL). (B) Principal component analysis (PCA) of targeted metabolite profiles showing group separation along the first two principal components (PC1 = 36.0%, PC2 = 19.7%). (C) Heatmap of the top 50 unique metabolites (row-*z*-scored) highlighting distinct metabolic signatures across groups. (D) Pathway enrichment analysis of significantly altered metabolites in BOS vs CTRL, BPF vs CTRL, and PFOS vs CTRL comparisons.

Heatmap clustering further emphasized these differences, revealing highly discriminative and coordinated metabolite abundance patterns across exposures (Figure 1C). Notably, subsets of metabolites exhibited opposing directions and magnitudes of change between chemicals, indicating distinct patterns of metabolic regulation rather than uniform up- or down-regulation. Volcano plots further illustrated the distribution, magnitude, and directionality of differential metabolites for each exposure, confirming that PFOS induced the broadest spread of significantly altered metabolites, whereas BOS and BPF produced comparatively more restricted profiles (Figure S1).

Consistent with these targeted findings, untargeted metabolomics analysis independently confirmed the presence of chemically distinct metabolic signatures (Figure S2). UpSet analysis of untargeted features demonstrated both shared and exposure-specific alterations, with a substantial number of metabolites uniquely perturbed by each chemical. Principal component analysis of untargeted profiles showed clear group separation, with PC1 and PC2 explaining 45.1% and 20.3% of the variance, respectively, reinforcing the strong exposure-driven structure of the metabolic data. Heatmap visualization of the top 50 unique metabolites further highlighted distinct and coordinated abundance patterns across exposure groups, mirroring the structured and chemical-specific responses observed in the targeted data set.

Pathway enrichment analysis further supported divergence between chemical exposures, revealing distinct patterns of enriched metabolic processes relative to controls (Figure 1D). Although several functional categories were represented across treatments, including pathways related to nucleotide metabolism, amino acid metabolism, and transport processes, the direction, significance, and enrichment strength differed between BOS, BPF, and PFOS. For instance, while tryptophan catabolism was highly activated in BOS treated embryos, and only moderately activated in BPF treated embryos, it was directionless in PFOS treated embryos. These results indicate that the chemical-specific metabolite signatures observed at the individual metabolite level are reflected in distinct patterns of pathway-level organization.

### Chemical Exposures Differentially Redirect Tryptophan Metabolism across Major Metabolic Branches

To further disentangle chemical-specific differences in tryptophan-related metabolites, the distribution of metabolite abundances across major metabolic branches was investigated (Figure 2). Heatmap visualization revealed clear separation of exposure groups driven by coordinated shifts in subsets of tryptophan-derived metabolites.

**Figure 2:**
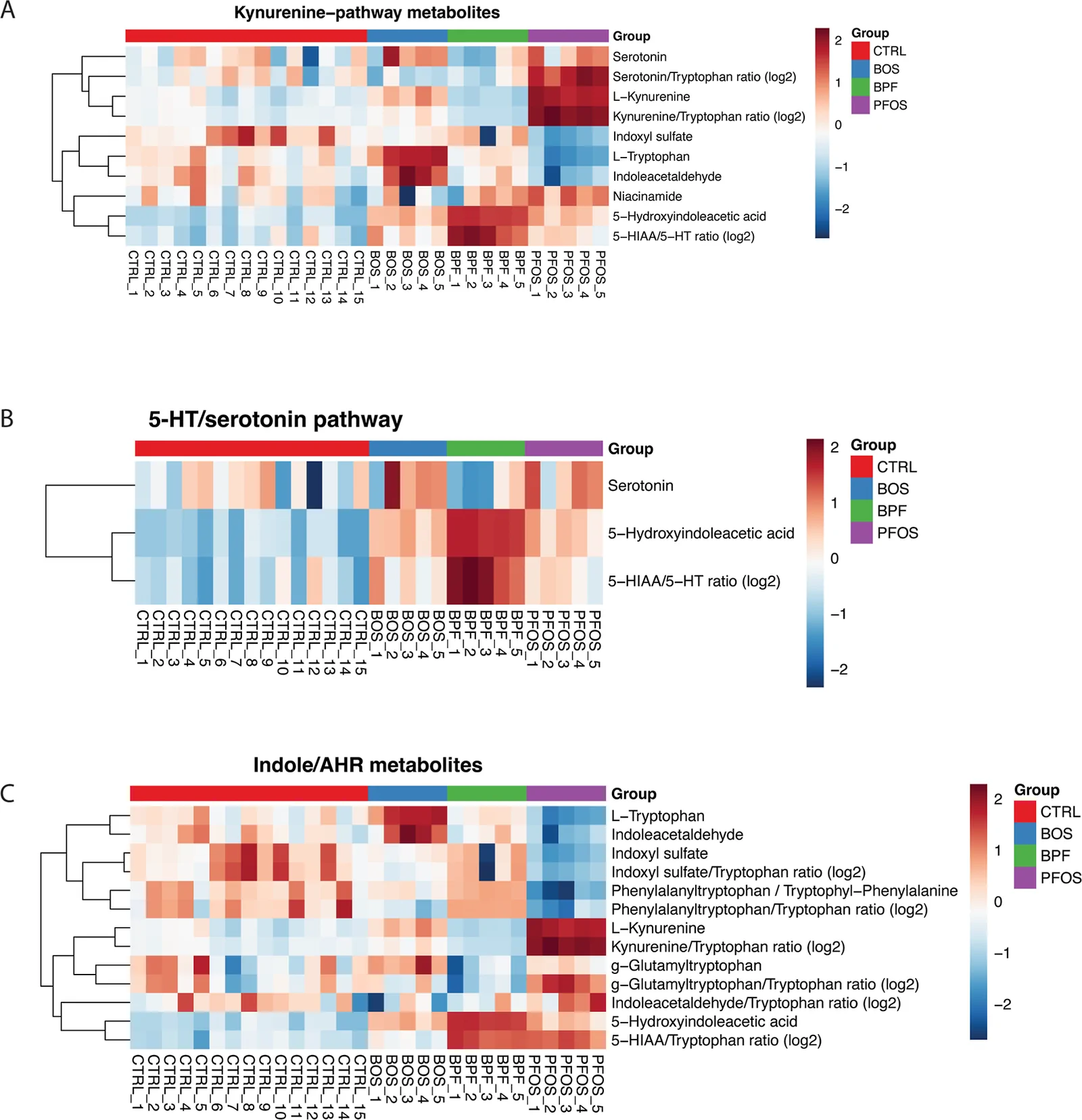
Exposure-specific modulation of tryptophan metabolism and downstream indole-AHR, kynurenine, and serotonin pathways. (A) Heatmap of kynurenine-pathway metabolites, including l-kynurenine, niacinamide, tryptophan-related measures, and selected indole/serotonin-related metabolites and log_2_ metabolite-to-tryptophan ratios, displayed as row *z*-scores across control (CTRL), BOS, BPF, and PFOS groups. (B) Heatmap of the 5-HT/serotonin pathway, showing serotonin, its primary catabolite 5-hydroxyindoleacetic acid (5-HIAA), and the log_2_(5-HIAA/5-HT) ratio across exposure groups. (C) Heatmap of indole and aryl hydrocarbon receptor (AHR)-related metabolites, including tryptophan-derived indoles and log_2_ metabolite-to-tryptophan ratios, across exposure groups. In all panels, columns represent individual samples and rows represent metabolites. Values are row *z*-scored, hierarchical clustering is applied to metabolites, and group membership is indicated by the annotation bar above each heatmap (CTRL, BOS, BPF, PFOS).

PFOS-treated embryos displayed higher relative levels of l-kynurenine together with reduced l-tryptophan, resulting in elevated kynurenine-to-tryptophan ratios consistent with increased flux through the kynurenine branch (Figure 2A). In contrast, BOS- and BPF-treated embryos showed comparatively stable kynurenine-to-tryptophan ratios, although both metabolites appeared moderately elevated in BOS samples. Niacinamide levels were also relatively higher in PFOS-treated embryos, suggesting altered downstream NAD^+^-related metabolism.

Metabolites within the 5-HT/serotonin branch displayed a distinct pattern, with elevated relative levels of 5-hydroxyindoleacetic acid (5-HIAA) in BPF-treated embryos. An increased log_2_(5-HIAA/5-HT) ratio in BPF samples was consistent with enhanced relative serotonin turnover compared to other exposure groups (Figure 2B). Within indole and microbiome-linked fates of tryptophan metabolism, indoleacetaldehyde and indoxyl sulfate showed comparatively lower levels in PFOS-exposed embryos (Figure 2C).

Together, these results indicate that early life chemical exposure is associated with branch-specific alterations in tryptophan metabolism, highlighting chemically distinct metabolic fingerprints that extend to subpathway-level organization within this central amino acid network.

### Coexpression Gene Modules Associate Differentially with Chemical Exposures and Linked Metabolic Signatures

Next, we used the transcriptomic data set from the three chemical exposures to construct modules of coexpressed genes using weighted correlation network analysis (WGCNA) (Figure 3). This framework enabled assessment of coordinated transcriptional responses and their relationships with metabolite variation. Network construction parameters were optimized by selecting a soft-thresholding power that achieved scale-free topology and stable connectivity (Supporting Information Figure S2).

**Figure 3:**
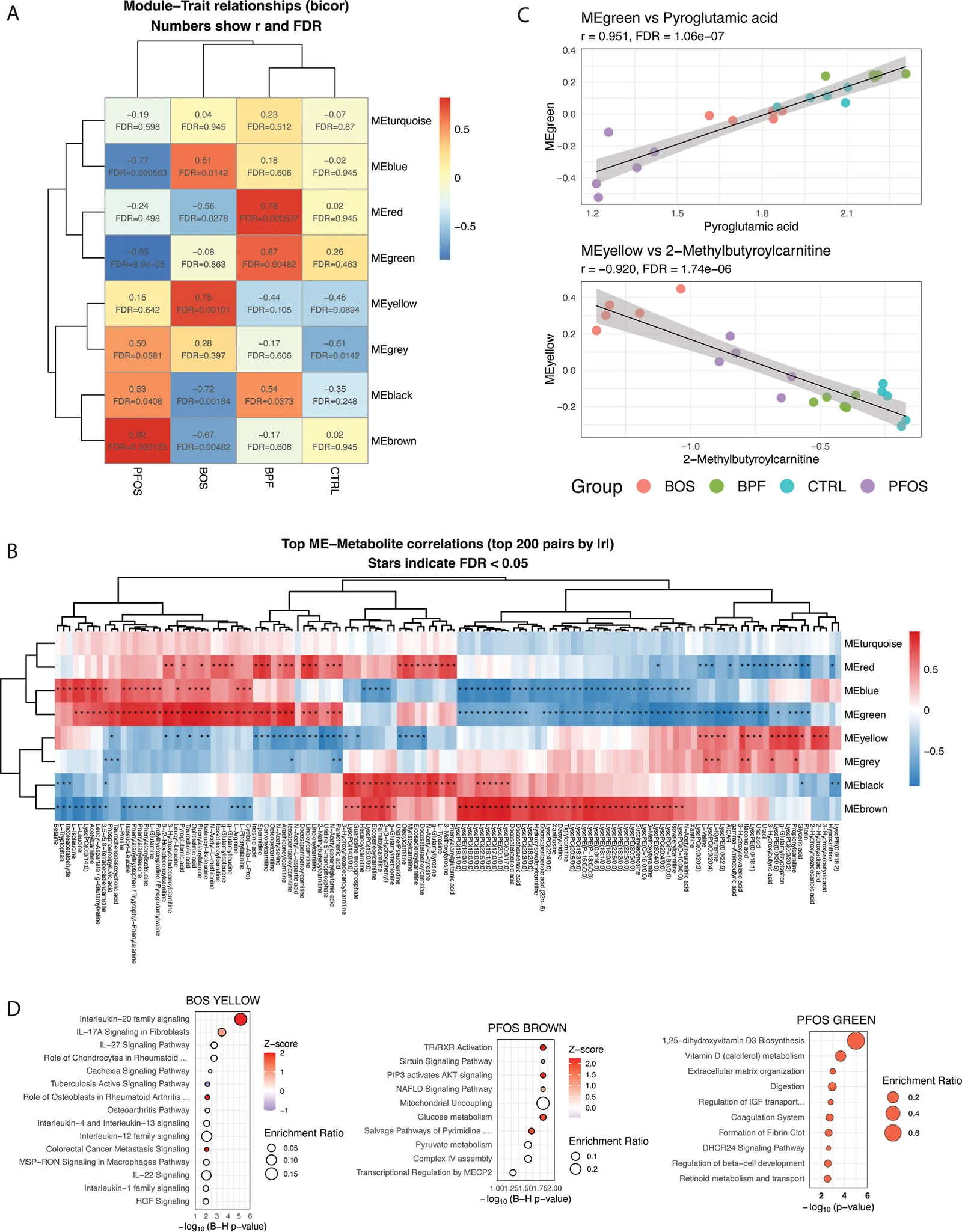
Weighted gene coexpression network analysis (WGCNA) links gene coexpression modules to chemical exposures, metabolites, and functional pathways. (A) Module–trait relationship heatmap showing biweight midcorrelation coefficients between module eigengenes and exposure groups. Each cell displays the correlation coefficient and FDR value, and colors indicate the direction and strength of association. (B) Heatmap of top-ranked module eigengene–metabolite associations based on correlations across exposure-group means. Associations are presented as exploratory because transcriptomic and metabolomic data sets were generated in independent but identically designed experiments. (C) Representative module–metabolite associations. Points represent exposure-group means, and regression lines are shown for visualization only. (D) Canonical pathway enrichment analysis of selected exposure-associated modules. The *x*-axis shows −log_10_(BH-adjusted *p*-values) for the BOS yellow and PFOS brown modules, whereas enrichment results for the PFOS green module are shown using nominal *p*-values. Point size indicates enrichment ratio, and color represents activation *Z*-score.

Module–trait analysis revealed exposure-specific associations between transcriptomic modules and chemical treatments (Figure 3A). PFOS showed a strong positive correlation with the brown module and additional positive associations with the black and gray modules, together with strong negative correlations with the green and blue modules. BOS was most strongly positively correlated with the yellow module, while showing negative correlations with the black and brown modules. BPF was strongly positively correlated with the red module and also positively correlated with the green and black modules. In contrast, CTRL showed fewer strong module relationships, with the most notable association being a negative correlation with the gray module. Together, these results indicate that chemical treatments preferentially perturbed coordinated transcriptional programs rather than isolated genes.

To integrate metabolomics with these transcriptional networks, metabolite abundances were correlated with transcriptomic module eigengenes. This analysis demonstrated coherent metabolite signatures across modules, with numerous significant module–metabolite relationships among the top-ranked correlations (Figure 3B). Notably, the PFOS-associated brown module, enriched for nuclear receptor signaling pathways, showed strong positive correlation with multiple phospholipid species, including lysophosphatidylethanolamines and lysophosphatidylcholines, while displaying predominantly negative correlations with amino acid metabolites. In contrast, the BOS-associated yellow module showed positive correlations with several amino acid-related metabolites, including kynurenine-associated signatures. Representative examples of these module–metabolites are shown in Figure 3C, including a strong positive association between MEgreen and pyroglutamic acid and a strong negative association between MEyellow and 2-methylbutyrylcarnitine, illustrating robust module–metabolite coupling across samples.

Functional enrichment analysis of exposure-associated modules revealed chemical-specific pathway patterns (Figure 3D). The BOS-associated yellow module was enriched for immune and cytokine signaling pathways, including IL-17A and IL-27 signaling, consistent with inflammatory transcriptional activation. The PFOS-associated brown module was enriched for nuclear receptor and metabolic pathways, including TR/RXR activation, sirtuin signaling, NAFLD signaling, and mitochondrial uncoupling. The PFOS-associated green module showed nominal enrichment (unadjusted *p*-values) for pathways related to vitamin D metabolism and extracellular matrix organization.

Together, these findings indicate that exposure-associated metabolic variation is organized within distinct transcriptional networks, linking chemical specific transcriptional responses with coordinated alteration in lipid, amino acid, and tryptophan metabolism.

### Host Microbial Status Modulates the Magnitude of Chemical-Induced Metabolic Perturbations

Principal component analysis of targeted metabolite profiles demonstrated that host microbial status influenced the separation of exposure groups, with WT and GF embryos forming partially distinct clusters within each chemical exposure (Figure 4A). Separation along the first principal component was driven primarily by chemical exposure, whereas microbial status introduced additional dispersion within exposure groups, indicating modulation of response magnitude without altering chemical-specific metabolic signatures. Heatmap analysis of the top discriminative metabolites further illustrated this relationship by resolving within-exposure differences between WT and GF embryos (Figure 4B). While samples clustered predominantly by exposure, microbiome-dependent shifts were reflected as distinct intensity patterns within individual chemical groups, highlighting genotype-dependent variation superimposed on stable chemical-specific profiles. Comparison of selected metabolites between WT and GF embryos illustrated representative microbiome-associated differences that varied by chemical exposure (Figure 4C). For several metabolites, including amino acids and redox-related compounds, GF embryos exhibited altered abundance relative to WT embryos, with the direction and magnitude of these differences depending on the specific chemical exposure.

**Figure 4:**
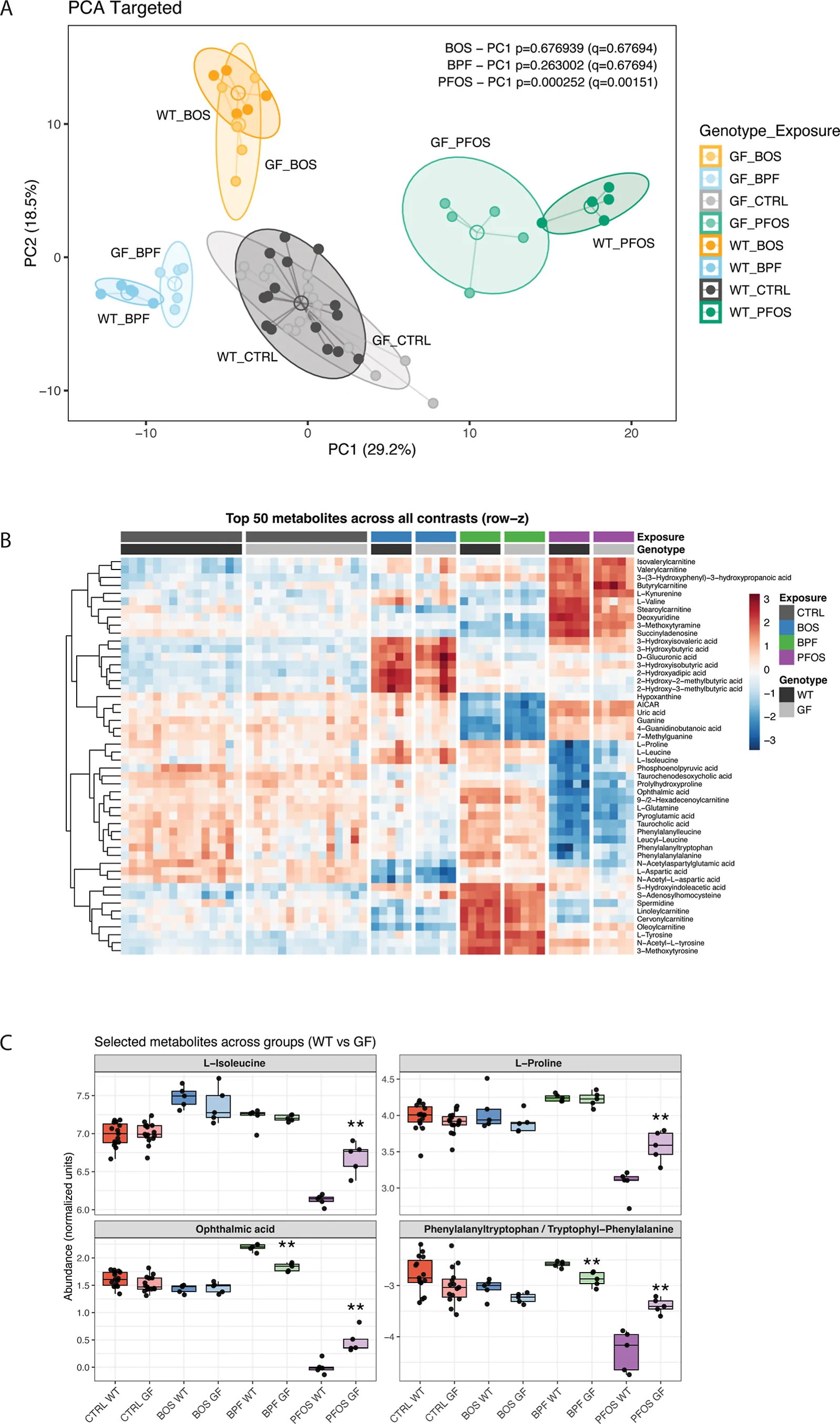
Microbiota-dependent metabolic responses revealed by targeted metabolomics. (A) Principal component analysis (PCA) of targeted metabolites demonstrating distinct clustering by microbial status (germ-free, GF; conventionally colonized, WT) and chemical exposure (BOS, BPF, PFOS, and CTRL). *p*-values for PC1 separation indicate exposure-specific effects within genotypes. (B) Heatmap of the top 50 metabolites across all contrasts, illustrating differential metabolic profiles between GF and WT zebrafish and among exposure groups. (C) Boxplots of selected metabolites showing genotype-dependent differences within each exposure group. Asterisks denote significant differences between WT and GF (*p < 0.05, **p < 0.01, ***p < 0.001; Wilcoxon test).

Untargeted metabolomics provided independent support for microbiome-dependent modulation of metabolic responses (Figure 5). Principal component analysis showed that chemical exposure remained the dominant source of variation, while microbial status introduced secondary separation within exposure groups, reinforcing that microbial effects primarily influence response magnitude rather than overall metabolic direction (Figure 5A). Heatmap analysis of the most discriminative features revealed similar stratification by microbial status, with coordinated differences in metabolite abundance between WT and GF embryos (Figure 5B). Notably, the extent of microbiome-associated separation varied across chemicals, with PFOS-exposed embryos showing the most pronounced differences between WT and GF conditions, whereas BOS and particularly BPF exhibited more limited divergence (Figure 5C).

**Figure 5:**
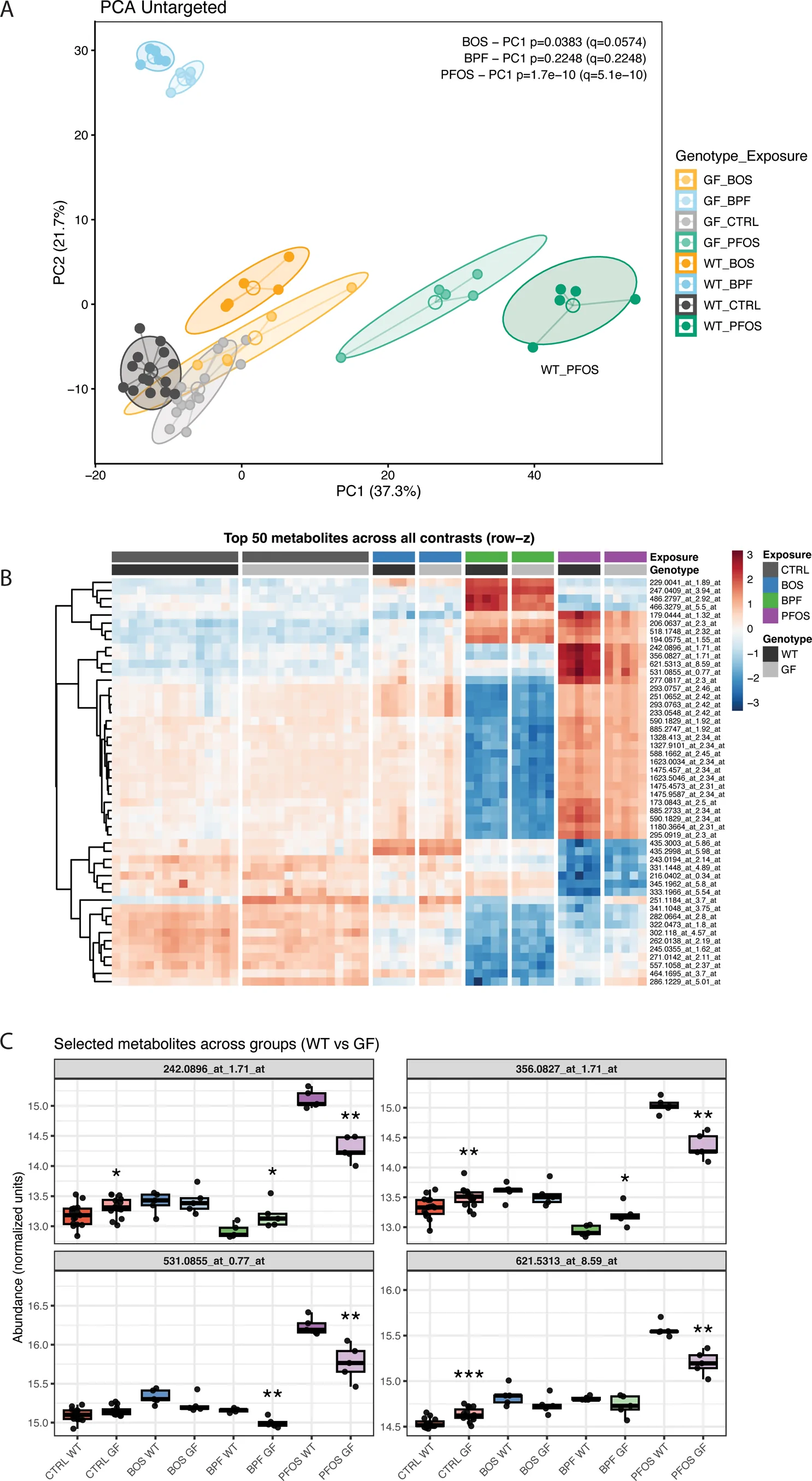
Microbiota-dependent metabolic responses revealed by untargeted metabolomics. (A) Principal component analysis (PCA) of untargeted metabolite profiles showing clear separation by microbial status (germ-free, GF; conventionally colonized, WT) and chemical exposure (BOS, BPF, PFOS, CTRL). PC1 and PC2 explain 37.3% and 21.7% of the variance, respectively. Reported *p*-values for PC1 indicate exposure-specific differences within genotypes. Ellipses represent group clustering. (B) Heatmap of the top 50 metabolites across all contrasts (row *z*-score normalized), illustrating distinct metabolic signatures between GF and WT zebrafish and across exposure groups. Samples are grouped by genotype and exposure. (C) Boxplots of selected metabolites highlighting genotype-dependent differences within each exposure group. Abundance is shown as normalized intensity. Asterisks indicate significant differences between WT and GF within the same exposure group (*p < 0.05, **p < 0.01, ***p < 0.001; Wilcoxon test).

Correlation analysis further identified significant associations between selected metabolites and individual bacterial amplicon sequence variants (ASVs), revealing structured patterns of coordinated positive and negative relationships rather than uniform microbiome-metabolite coupling (Supporting Information Figure S4). Several ASVs showed positive associations with metabolites linked to amin acid turnover, mitochondrial fatty acid metabolism, bile acid metabolism, and oxidative stress adaptation, including acylcarnitines, ophthalmic acid, glutamine, and branched-chain amino-acid related metabolites. In contrast, other ASVs displayed predominantly negative correlations with these metabolic features, indicating distinct microbial associations with exposure-associated metabolic states. Together, these analyses indicate that microbial colonization status modulates developmental metabolic responses while preserving overall chemical-specific metabolic fingerprints.

### Germ-Free Status Reveals Chemical-Specific Modulation of Metabolite Responses

To directly assess how host microbial status modifies chemical-induced targeted metabolic perturbations, we compared metabolite abundances between germ-free (GF) and conventionally colonized wild-type (WT) embryos within each exposure group (Figure 6). This within-exposure comparison isolates microbiota-dependent effects while controlling for chemical-specific metabolic signatures.

**Figure 6:**
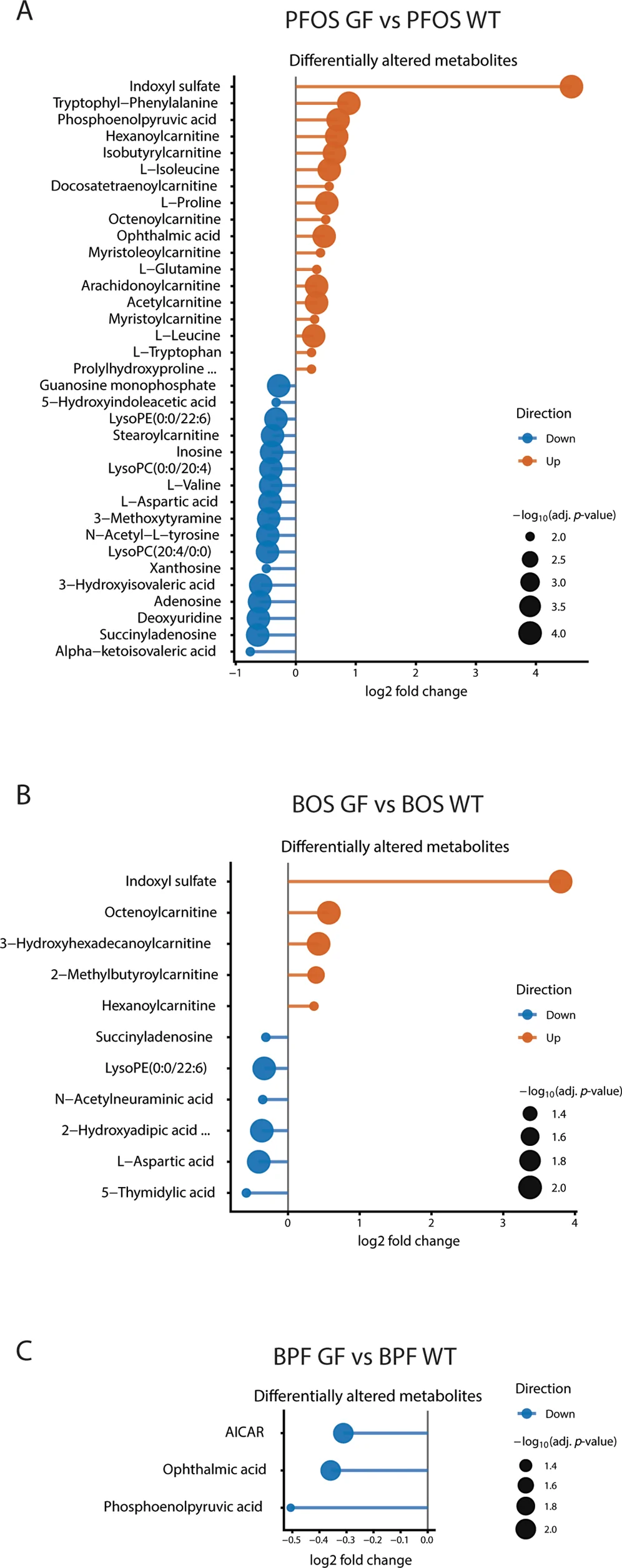
Differential metabolite responses in germ-free versus conventional zebrafish following chemical exposure. (A) Forest plot of differentially altered metabolites in PFOS-exposed germ-free zebrafish (PFOSGF) compared with PFOS-exposed conventional zebrafish (PFOSWT), shown as log_2_ fold change relative to PFOSWT. (B) Corresponding forest plot for BOS-exposed germ-free (BOSGF) versus BOS-exposed conventional zebrafish (BOSWT). (C) Forest plot for BPF-exposed germ-free (BPFGF) versus BPF-exposed conventional zebrafish (BPFWT). Color denotes direction of change (orange, increased; blue, decreased in germ-free relative to conventional zebrafish). Only metabolites passing the adjusted significance threshold are shown.

PFOS exposure showed the most extensive microbiota-dependent differences, with a broad set of metabolites displaying altered abundance in GF embryos relative to WT counterparts (Figure 6A). Metabolites related to amino acid metabolism, acylcarnitine profiles, nucleotide turnover, membrane lipid metabolism and tryptophan-associated pathways were prominently altered, indicating widespread metabolic remodeling in the absence of host-associated microbial communities. Several acylcarnitines and amino-acid related metabolites, including indoxyl sulfate, accumulated in PFOS-exposed GF embryos, consistent with disrupted mitochondrial stress adaptiation and altered tryptophan metabolism. In parallel, multiple nucleotide-associated metabolites and lysophospholipid species were reduced, suggesting impaired cellular maintenance and membrane remodeling capacity. Together with our previous observations of increased PFOS-induced developmental toxicity and edema in microbiota-depleted embryos,^15^ these findings support the interpretation that microbial colonization contributes to metabolic resilience during PFOS-induced chemical stress.

In contrast, BOS exposure resulted in a more restricted set of microbiota-dependent metabolic differences (Figure 6B). GF embryos showed increased abundance of indoxyl sulfate and selected acylcarnitines, suggesting altered tryptophan metabolism and mitochondrial fatty acid handling in the absence of host-associated microbial communities. Several metabolites related to nucleotide metabolism, amino acid metabolism, and membrane lipid remodeling, including succinyladenosine, thymidylic acid, aspartic acid, and LysoPE, were reduced in GF embryos relative to WT counterparts. Notably, our previous work demonstrated that microbiota-depleted embryos are less sensitive to BOS-induced developmental toxicity,^15^ indicating that microbial colonization may contribute to BOS-associated stress responses rather than confer metabolic resilience, as observed for PFOS. These findings suggest that microbiota-dependent modulation of chemical stress responses is compound-specific and may either attenuate of potentiate developmental toxicity depending on the exposure content.

For BPF exposure, microbiota-dependent differences were comparatively limited, with only a small number of metabolites showing significant changes between GF and WT embryos (Figure 6C). The affected metabolites included AICAR, ophthalmic acid, and phosphoenolpyruvic acid, implicating altered energetic stress signaling, redox metabolism, and glycolytic intermediate metabolism. Compared with WT embryos, GF embryos displayed lower levels of these metabolites, suggesting an attenuated metabolic stress response in the absence of host-associated microbial communities. Together with our previous findings demonstrating increased BPF toxicity in microbiota-depleted embryos,^15^ these results support the interpretation that microbial colonization contributes to adaptive metabolic resilience during chemical stress.

Together, these comparisons demonstrate that host microbial status modulates chemical-induced metabolic responses in a compound-specific manner. PFOS exposure showed the strongest microbiota-dependent metabolic remodeling, characterized by broad disruption of amino acid, lipid, nucleotide, and mitochondrial-associated metabolism in GF embryos. BOS exposure displayed more selective microbiota-dependent effects and, notably, an opposite toxicity pattern in which microbial colonization appeared to enhance rather than buffer developmental stress responses. In contrast, BPF-induced metabolic differences were modest but consistent with impaired adaptive stress signaling in microbiota depleted embryos. Collectively, these findings indicate that host-associated microbial communities function as contextual modifiers of developmental metabolic adaption during chemical stress, with effects that differ fundamentally depending on the chemical exposure.

## Discussion

Understanding how environmental chemicals disrupt metabolism during early development is critical, as metabolic perturbations at this stage can precede macroscopic changes and influence later-life physiological outcomes.^1,3^ A wide range of environmental contaminants, including PFAS, bisphenols, and agricultural fungicides, have been associated with metabolic dysfunction in both experimental models and human populations.^22^ However, much of the existing literature has focused on individual compounds or isolated end points,^23^ leaving unresolved whether early metabolic disruption represents a shared stress response or instead reflects distinct, compound-specific toxicodynamic patterns. Addressing this gap requires approaches that move beyond single biomarkers to capture coordinated metabolic changes across pathways.

Here, we applied targeted and untargeted metabolomic profiling in zebrafish embryos to resolve chemical-specific metabolic responses to PFOS, BPF, and BOS during early development. Our results show that early life metabolic disruption differed markedly between compounds rather than following a uniform pattern across toxicants. The novelty of the present study lies in the combined use of pathway-focused and discovery-based metabolomics to characterize biochemical responses across central metabolism, together with the inclusion of both germ-free and conventionally colonized embryos to assess how microbial status modifies chemical-induced metabolic perturbations. These findings extend current knowledge on the metabolic effects of PFAS, including PFOS,^24^ bisphenols,^25^ and mitochondrial toxicants,^26^ by demonstrating that these chemicals elicit distinct and structured metabolic phenotypes during early development. Importantly, the targeted and untargeted metabolomics data sets provided complementary support for this conclusion. Untargeted profiles independently recapitulated the exposure-specific separation observed in the targeted data set and revealed additional features contributing to compound-specific metabolic signatures. This concordance across analytical platforms strengthens the robustness of the observed metabolic phenotypes and supports the conclusion that early life chemical exposure induces distinct metabolic programs rather than a generalized stress response.

Per- and polyfluoroalkyl substances (PFAS), including PFOS, have been widely reported to disrupt lipid and energy metabolism, impair mitochondrial function, and alter bile acid and amino acid homeostasis in mammalian and aquatic models.^27–29^ Consistent with these observations, PFOS elicited the most extensive metabolic perturbation in our study. A significant decrease in amino acid levels was observed relative to controls, with particularly pronounced reductions in l-glutamine and pyroglutamic acid. During normal zebrafish development, embryos between 72 and 96 hpf exhibit elevated l-glutamine levels^8^ and increased glutamine catabolism to meet the heightened metabolic demands associated with rapid cell proliferation and differentiation. In contrast, PFOS exposure in our study resulted in a marked downregulation of glutamine levels, suggesting a disruption of glutamine homeostasis. Given the central role of glutamine as a nitrogen donor in de novo nucleotide biosynthesis,^30^ this perturbation may indicate impaired nucleotide synthesis and, consequently, altered cellular proliferation and developmental processes. As tryptophan metabolism integrates immune, redox, and xenobiotic-responsive signaling pathways during early development, the observed shifts in indole and kynurenine metabolites may reflect chemical-specific rerouting of ligand pools linked to aryl hydrocarbon receptor (AHR)-associated signaling rather than uniform metabolic depletion.

In contrast, BOS produced a more restricted metabolic profile, consistent with previous evidence that succinate dehydrogenase-targeting fungicides primarily affect mitochondrial respiration and associated metabolic pathways.^31^ Embryos exposed to BOS exhibited elevated levels of β-hydroxybutyric acid, hydroxy-2-methylbutyric acid, hydroxyisobutyric acid, d-gluconic acid, and selected short- and medium-chain acylcarnitines, together with increased succinyladenosine. The increase in β-hydroxybutyrate, a key ketone body, suggests a metabolic shift toward fatty acid-derived energy substrates during stress,^32^ potentially reflecting altered mitochondrial efficiency or redox balance. Concurrent elevations in hydroxy-2-methylbutyric and hydroxyisobutyric acids,^33^ metabolites associated with branched-chain amino acid catabolism, together with accumulation of acylcarnitine species, are compatible with altered β-oxidation and incomplete mitochondrial processing. Increased d-gluconic acid further suggests enhanced flux through the pentose phosphate pathway, potentially reflecting oxidative stress-related metabolic adaptation.^34^ Consistent with these alterations in energy and redox metabolism, concurrent reductions in aspartate, *N*-acetylaspartate, and 2-hydroxyadipic acid indicate targeted effects on amino acid-associated pathways. Decreased levels of *N*-acetylneuraminic acid and *N*-acetylaspartate—metabolites essential for neuronal development, membrane stability, and neuron-glia metabolic coupling^35,36^ —are consistent with perturbation of neurodevelopmentally relevant metabolic pathways during early development.

BPF, a structural analog of bisphenol A, has been linked to endocrine, neurochemical and liver metabolism alterations in prior studies,^37,38^ and the distinct metabolic profile observed here further supports the notion that bisphenols perturb metabolism through mechanisms that differ from both PFAS and BOS. Notably, BPF-exposed embryos exhibited increased levels of l-tyrosine and its methylated derivative 3-methoxytyrosine, indicating altered aromatic amino acid metabolism and potential disruption of tyrosine-derived signaling pathways. Concurrent elevations in linoleylcarnitine, oleoylcarnitine, and isocatrienoylcarnitine suggest impaired mitochondrial β-oxidation and accumulation of long-chain fatty acid intermediates. The buildup of multiple acylcarnitine species is indicative of mitochondrial inefficiency or overload.^39^ Together, these findings point to BPF-induced reprogramming of energy metabolism, characterized by disrupted fatty acid oxidation and secondary perturbations in neurotransmitter-related amino acid metabolism.

To place these metabolite-level differences into a systems-level regulatory context, the chemical-specific metabolic phenotypes observed here were further examined through coordinated transcriptional network responses. Network-level analysis of transcriptomic data revealed gene coexpression modules whose eigengenes were differentially associated with PFOS, BOS, and BPF. Correlating metabolite abundances with transcriptomic module eigengenes enabled integration of exposure-associated metabolic patterns with underlying transcriptional programs at the level of coordinated responses, rather than implying matched individual-level covariance. Functional enrichment of exposure-associated modules revealed that PFOS-correlated modules were enriched for nuclear receptor and metabolic pathways, including TR/RXR activation and sirtuin signaling, whereas BOS-correlated modules were enriched for immune and cytokine signaling pathways such as IL-17A and IL-27 signaling. In addition, the positive association between kynurenine abundance and the BOS-associated yellow module further links activation of inflammatory transcriptional programs with coordinated shifts in tryptophan metabolism. The PFOS-associated green module also showed nominal enrichment for pathways related to vitamin D metabolism and extracellular matrix organization and should therefore be interpreted cautiously. These findings indicate that the structured metabolic fingerprints observed at the metabolite level reflect higher-order, chemically specific transcriptional network organization, consistent with network-based and multiomics integration approaches used to resolve coordinated biological responses to environmental stressors.^18,40,41^

Beyond host transcriptional regulation, comparison of germ-free and conventionally colonized embryos showed that microbial status modulated the magnitude of chemical-induced metabolic perturbations while preserving exposure-specific clustering patterns. This is consistent with previous studies showing that the microbiome can influence xenobiotic metabolism, nutrient availability, and host energy balance during early life.^42–44^ Our data therefore support the interpretation that microbial status acts as a contextual modifier of host sensitivity to chemical stress, rather than as a primary determinant of compound-specific metabolic outcomes.

Importantly, microbiome-dependent modulation differed substantially across chemicals. PFOS exposure showed extensive microbiota-dependent metabolic remodeling, together with increased developmental toxicity and edema in microbiota depleted embryos,^15^ supporting a protective role of microbial colonization during PFOS-induced stress. Similarly, although microbiota-dependent metabolic differences were comparatively limited for BPF, reduced levels of stress-associated metabolites in germ-free embryos together with increased developmental toxicity^15^ suggest impaired adaptive stress signaling in the absence of microbial communities. In contrast, microbiota depletion reduced sensitivity to BOS-induced developmental toxicity,^15^ indicating that microbial colonization may also contribute to or amplify specific chemical stress responses depending on the exposure context.

These findings suggest that microbiota-dependent effects cannot be interpreted as uniformly protective or uniformly sensitizing. Rather, host-associated microbial communities appear to influence how developing embryos adapt metabolically to chemical stress, with the direction and magnitude of this modulation varying across compounds and mechanisms of action. Importantly, the overall chemical-specific metabolic fingerprints remained preserved across microbial conditions, indicating that microbiota primarily influences response magnitude, adaptive capacity, and susceptibility rather than reversing the core metabolic identity of chemical exposure.

This distinction is relevant for interpretation and regulatory application. The findings do not imply that germ-free embryos are required for routine hazard identification, since the direction and compound specificity of metabolic responses were largely preserved in conventionally colonized embryos. Instead, the germ-free comparison highlights that microbial status may influence effect magnitude, susceptibility, benchmark-response estimates, and biomarker intensity under otherwise identical exposure conditions.

This study was designed to resolve early, system-level metabolic responses to environmental chemicals under controlled developmental and microbial conditions. By combining zebrafish embryos, targeted and untargeted metabolomics, and transcriptomic network analysis at 5 dpf, we captured coordinated metabolic perturbations during a defined window of early life chemical stress. However, this design does not address temporal dynamics, tissue-specific adaptations, later-life outcomes, or causal microbial–metabolite interactions. Because the omics data sets were generated from independent but identically designed exposure experiments, cross-omics associations should be interpreted as coordinated group-level responses rather than matched individual-level covariance. The translational interpretation of these microbiome-dependent effects should also account for host and microbiome specificity. Zebrafish embryos provide a controlled vertebrate model for identifying principles of microbiome-dependent modulation and chemical-specific metabolic stress, but their early life microbiota, gut maturation, and environmental microbial exposures differ from mammalian and human systems. Thus, the model should not be viewed as a direct surrogate for human microbiome composition or exposure scenarios bur rather as an indication of potential detrimental effects on conserved pathways.

## Conclusions

Within this controlled experimental framework, the present findings support a model in which chemical mode of action primarily defines the direction and structure of metabolic perturbation, whereas host microbial status modulates the magnitude and resolution of these responses. Across exposures, PFOS, BOS, and BPF elicited distinct metabolic programs that aligned with exposure-associated transcriptional network signatures, indicating that early life chemical stress does not converge on a uniform metabolic response but instead reflects compound-specific network organization. Within this framework, the microbiome functions as a contextual modifier that scales metabolic response intensity without overriding chemical specificity, consistent with the observation that germ-free status altered effect size rather than pathway directionality. The gradient of microbiome dependence observed across chemicals further suggests that microbial modulation becomes most apparent when chemical-induced metabolic burden exceeds a threshold, providing a conceptual basis for understanding variability in developmental susceptibility to environmental exposures.

Overall, these findings demonstrate that early life exposure to environmental chemicals induces distinct metabolic stress phenotypes shaped, but not determined, by host microbial status. By resolving chemical-specific metabolic disruption while accounting for contextual modifiers, metabolomic approaches provide a valuable framework for improving mechanistic interpretation of developmental toxicity.^7^ Such strategies complement existing toxicological paradigms by capturing early biochemical liabilities and sources of variability in chemical susceptibility, thereby contributing to more nuanced and biologically grounded assessments of early life chemical effects.^45^ Together, these findings highlight that early life metabolic toxicity is not a uniform response to environmental chemicals but instead reflects compound-specific metabolic network perturbations whose magnitude can be shaped by host microbial context.

## Supplementary Material



## Notes

Additional experimental details for targeted and untargeted metabolomics, compound exposure conditions, metabolite annotation and quality-control procedures; supplementary tables of quantified metabolites and untargeted features; supplementary figures of targeted and untargeted metabolomic profiles, transcriptomic module analyses, and microbiome–metabolite associations (PDF)
