# Supplementary material for: Chemical-Specific Metabolic Disruption during Early Zebrafish Development is Modulated by the Microbiome
Source: Environ Health (Wash). 2026 Jul 10;4(10):2401–15. doi: 10.1021/envhealth.6c00215 (PMC13681445; doi:10.1021/envhealth.6c00215)
Supplement: Supplementary file 1 [file eh6c00215_si_001.pdf]

Supporting Information

Chemical-specific metabolic disruption during early zebrafish development is modulated by the microbiome

Nanna Fyhrquist[1,3*], Lydia Gugescu[1], Emma Wincent[1], Harri Alenius[1,2*]

1 Institute of Environmental Medicine, Karolinska Institutet, Stockholm, Sweden
2 Human Microbiome Research Program (HUMI), University of Helsinki, Helsinki, Finland
3 Department of Health Sciences, Karlstad University, Karlstad, Sweden

*Correspondence:
Nanna Fyhrquist – Department of Health Sciences, Karlstad University, Karlstad, Sweden
E-mail address: nanna.fyhrquist@ki.se (N. Fyhrquist).

Harri Alenius – Institute of Environmental Medicine, Karolinska Institutet, Stockholm, Sweden. E-mail address: harri.alenius@ki.se (H. Alenius).

Metabolomics analysis in detail

Sample preparation was performed according to A et al. [1] with minor modifications. Briefly, 500 µL extraction buffer (80/20 v/v methanol:water) containing internal standards for both GC–MS and LC–MS was added to the tubes together with one tungsten bead. Samples were shaken at 30 Hz for 3 minutes in a mixer mill, followed by centrifugation at 4 °C, 14,000 rpm for 10 minutes. The supernatant was transferred to microvials, evaporated to dryness in a speed-vac concentrator, and stored at −80 °C until further analysis.

Boscalid, PFOS, and BPF standards were diluted in extraction buffer to a stock concentration of 250 ng/µL. Small aliquots of the supernatants were pooled to generate quality control (QC) samples. MS/MS analysis (LC–MS) was performed on the QC samples for metabolite identification. All samples were analyzed in randomized run order on both GC–MS and LC–MS platforms.

GC–MS analysis

Derivatization and GC–MS analysis were performed as previously described [2]. A 0.5 µL aliquot of the derivatized sample was injected in splitless mode using an L-PAL3 autosampler (CTC Analytics AG, Switzerland) into an Agilent 7890B gas chromatograph equipped with a 10 m × 0.18 mm fused silica capillary column with a chemically bonded 0.18 µm Rxi-5 Sil MS stationary phase (Restek Corporation, USA).

The injector temperature was set to 270 °C. The purge flow rate was 20 mL min^{-1}, with purge activated after 60 seconds. The carrier gas flow through the column was 1 mL min^{-1}. The column temperature was held at 70 °C for 2 minutes, increased by 40 °C min^{-1} to 320 °C, and held for 2 minutes.

The column effluent was introduced into the ion source of a Pegasus BT time-of-flight mass spectrometer (GC/TOFMS; Leco Corp., St Joseph, MI, USA). The transfer line and ion source temperatures were 250 °C and 200 °C, respectively. Ions were generated using a 70 eV electron beam at an ionization current of 2.0 mA, and 30 spectra s^{-1} were recorded over a mass range of m/z 50–800. The acceleration voltage was applied after a solvent delay of 150 seconds. Detector voltage ranged from 1800–2300 V.

Boscalid, PFOS, and BPF standards were derivatized in the same manner as the samples, and 20 ng were injected onto the column.

LC–MS analysis

Prior to LC–MS analysis, samples were re-suspended in 10 µL methanol and 10 µL water. All samples were first analyzed in positive ion mode, after which the instrument was switched to negative ion mode and a second injection of each sample was performed.

Chromatographic separation was performed on an Agilent 1290 Infinity UHPLC system (Agilent Technologies, Waldbronn, Germany). A 2 µL aliquot of each sample was injected onto an Acquity UPLC HSS T3 column (2.1 × 50 mm, 1.8 µm C18) combined with a 2.1 × 5 mm, 1.8 µm VanGuard precolumn (Waters Corporation, Milford, MA, USA) maintained at 40 °C.

The gradient elution buffers were A (H_2O with 0.1% formic acid) and B (75/25 acetonitrile:2-propanol with 0.1% formic acid), with a flow rate of 0.5 mL min^{-1}. The gradient consisted of 0.1–10% B over 2 minutes, increased to 99% B over 5 minutes and held for 2 minutes. B was then reduced to 0.1% for 0.3 minutes, the flow rate increased to 0.8 mL min^{-1} for 0.5 minutes, held for 0.9 minutes, and finally returned to 0.5 mL min^{-1} for 0.1 minutes before the next injection.

Compounds were detected using an Agilent 6546 Q-TOF mass spectrometer equipped with a Jet Stream electrospray ion source operating in either positive or negative ion mode. Instrument settings were identical between ionization modes except for the capillary voltage polarity.

A reference interface was used for accurate mass calibration. Reference ions purine (4 µmol/L) and HP-0921 (Hexakis(1H,1H,3H-tetrafluoropropoxy)phosphazine) (1 µmol/L) were infused into the MS at a flow rate of 0.05 mL min^{-1} for internal calibration. The monitored ions were purine m/z 121.05 and 119.03632, and HP-0921 m/z 922.0098 and 966.000725 for positive and negative ion mode, respectively.

The gas temperature was 150 °C, drying gas flow 8 L min^{-1}, and nebulizer pressure 35 psig. The sheath gas temperature was 350 °C with a flow of 11 L min^{-1}. The capillary voltage was set to 4000 V in both positive and negative ion modes. The nozzle voltage was 300 V, fragmentor voltage 120 V, skimmer 65 V, and OCT 1 RF Vpp 750 V. Collision energy was set to 0 V. Data were acquired in centroid mode over an m/z range of 70–1700 with an acquisition rate of 4 scans s^{-1} (1977 transients per spectrum).

Boscalid, PFOS, and BPF standards were diluted in 80/20 v/v methanol:water, and 5 ng were injected onto the column.

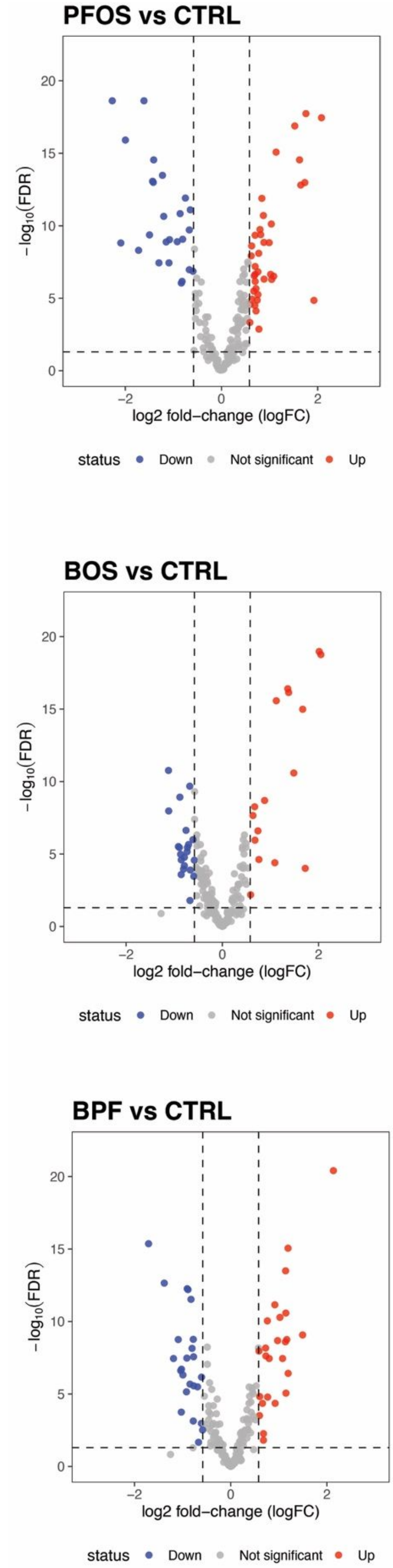

Figure S1. Volcano plots of differential metabolites following PFOS, BOS, and BPF exposure compared with control. Each point represents a detected metabolite. The x-axis shows log2 fold-change (logFC), and the y-axis shows –log10 adjusted *p*-value (FDR). Dashed vertical lines indicate the fold-change threshold ($|\log2FC| \geq 0.58$, corresponding to ~1.5-fold change), and the horizontal dashed line indicates the statistical significance threshold ($FDR < 0.05$). Metabolites meeting both criteria are colored red (upregulated) or blue (downregulated), while non-significant metabolites are shown in grey.

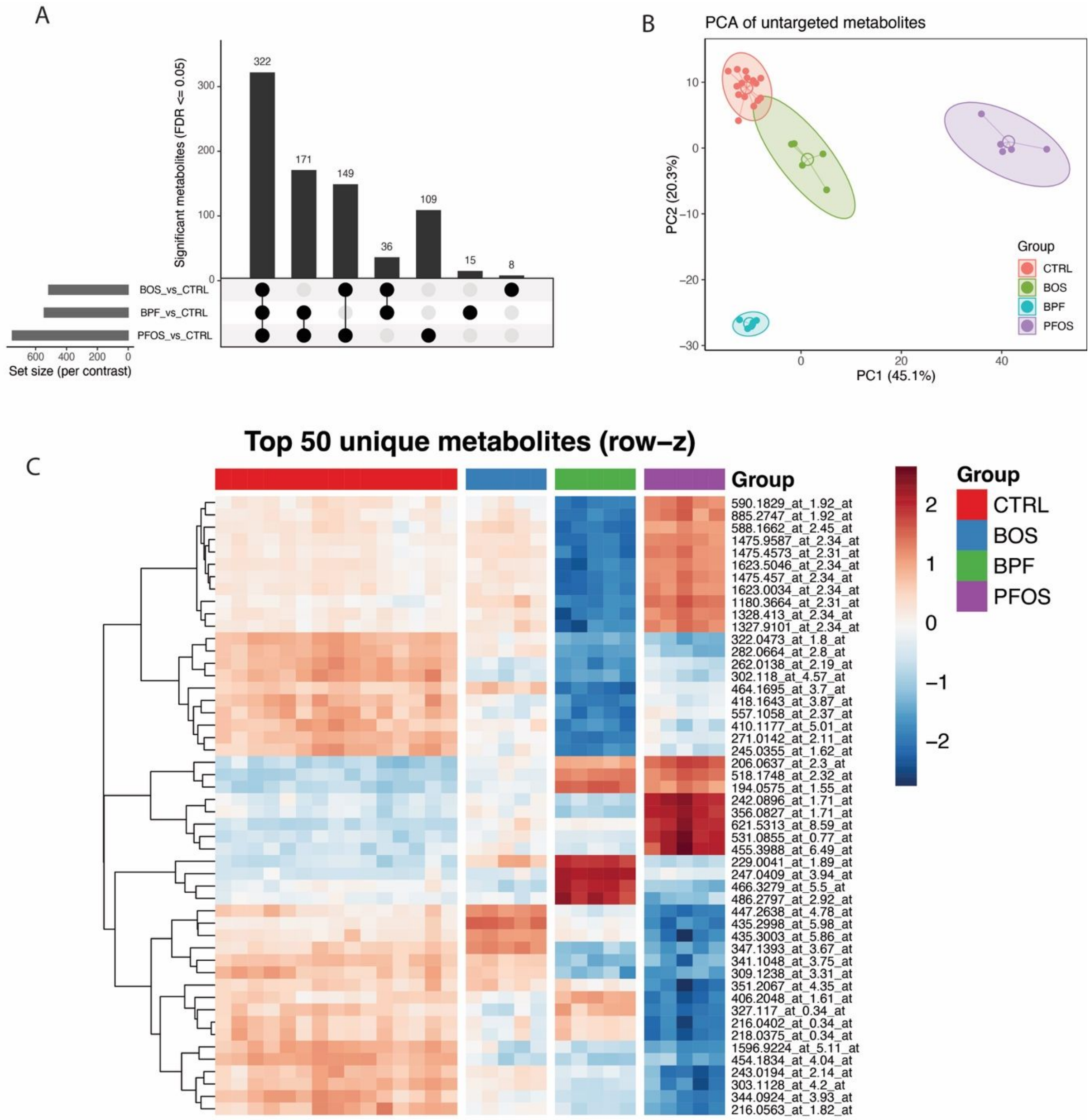

Figure S2. Untargeted metabolomics reveals distinct metabolic alterations following chemical exposure. (A) UpSet plot showing the overlap of significantly altered metabolites (FDR < 0.05) across exposure groups compared with control (CTRL). Bar plots indicate the number of significant metabolites per contrast, and connected dots denote shared and unique features among BOS, BPF, and PFOS exposures. (B) Principal component analysis (PCA) of untargeted metabolite profiles demonstrating separation between exposure groups (CTRL, BOS, BPF, PFOS). PC1 and PC2 explain 45.1% and 20.3% of the variance, respectively. Ellipses indicate group clustering. (C) Heatmap of the top 50 unique metabolites (row z-score normalized), highlighting distinct metabolic signatures across exposure groups. Samples are grouped by treatment, and metabolites are hierarchically clustered.

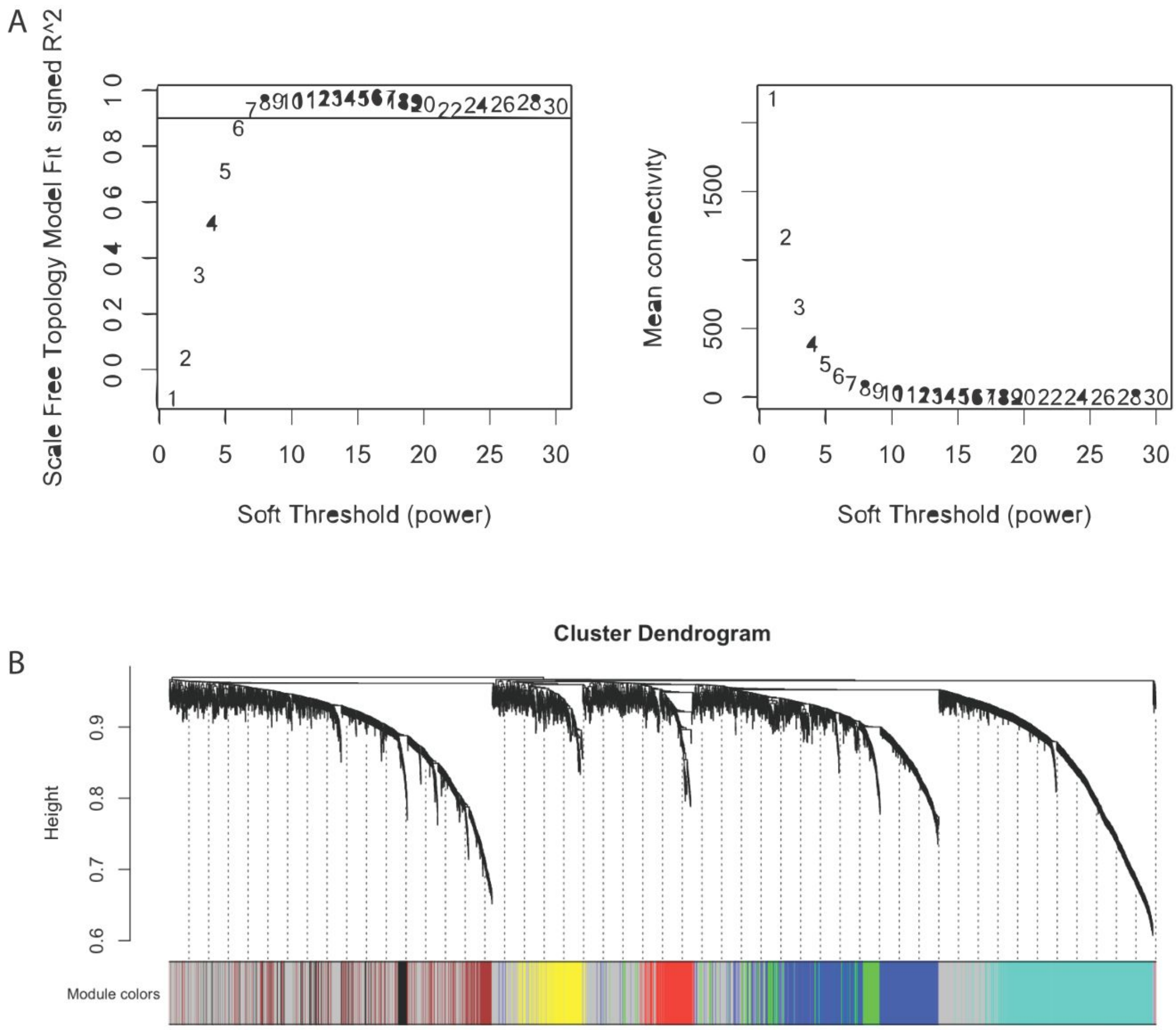

Supplementary Figure S3. Determination of soft-thresholding power and module identification in weighted correlation network analysis (WGCNA). A) Selection of soft-thresholding power based on the scale-free topology fit index (left) and mean connectivity (right). A power of 6 was chosen to achieve approximate scale-free topology ($R^2 > 0.9$). (B) Hierarchical clustering dendrogram of genes showing WGCNA module assignments by dynamic tree cutting. Each color represents a distinct gene module identified in the WGCNA.

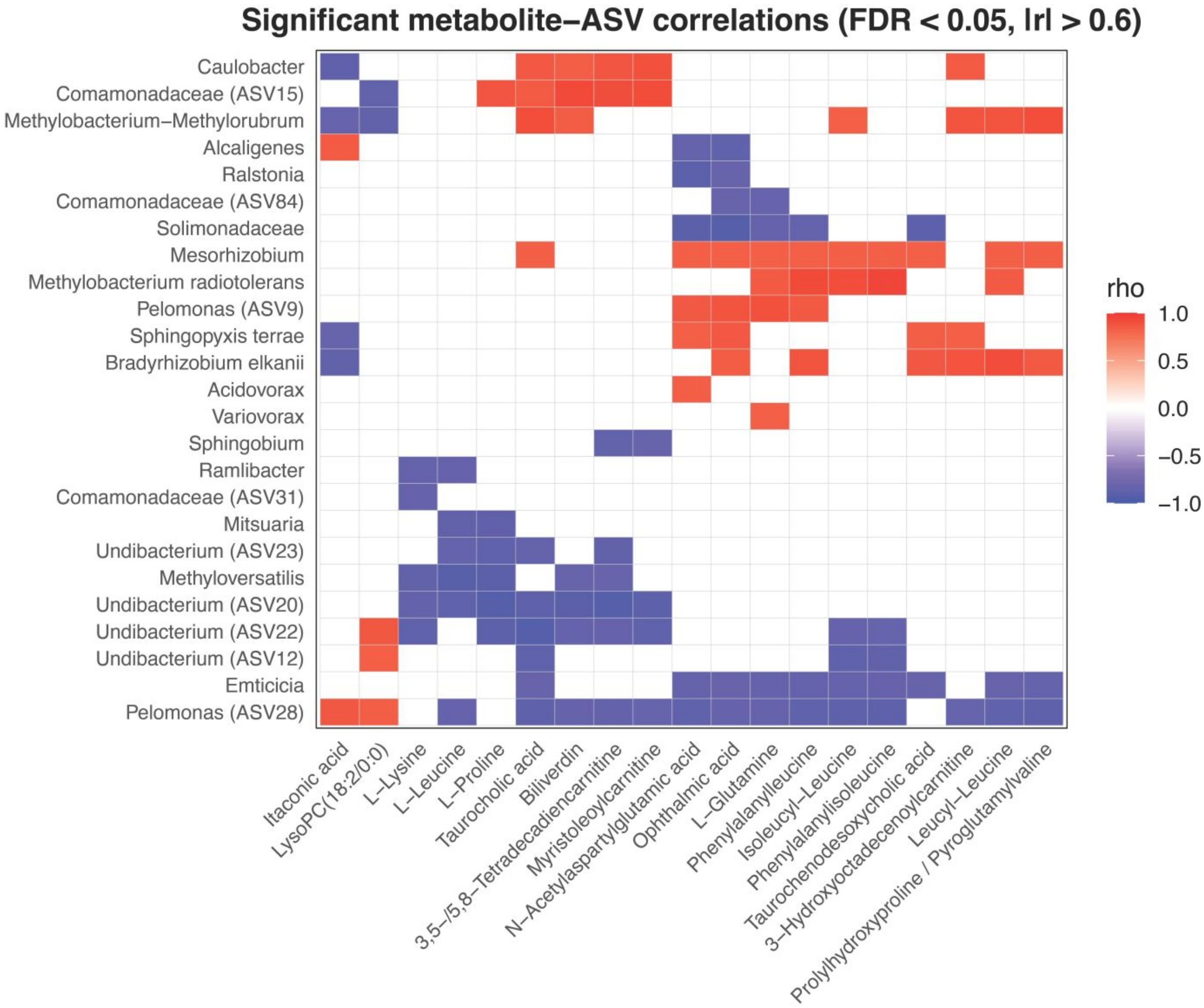

Supplementary Figure S4. Correlation between metabolites and bacterial ASVs. Heatmap showing significant metabolite–ASV correlations (FDR < 0.05 and |ρ| > 0.6). Rows represent bacterial amplicon sequence variants (ASVs) and columns represent metabolites. The color scale indicates the direction and strength of the Spearman correlation coefficient (ρ), with red indicating positive correlations and blue indicating negative correlations.

Supplementary Table S1

metabolite_names	HMDB	KEGG
2-/3-Hydroxymyristoylcarnitine	HMDB0013166	
2-Hydroxy-2-methylbutyric acid	HMDB0001987	
2-Hydroxy-3-methylbutyric acid	HMDB0000407	
2-Hydroxyadipic acid / 3-Hydroxyadipic acid / 3-Hydroxymethylgl	HMDB0000321	C02360
2-Hydroxyhexadecanoic acid	HMDB0031057	
2-Methylbutyroylcarnitine	HMDB0000378	
2-Methylbutyrylglycine	HMDB0000339	
2'-Deoxyguanosine 5'-monophosphate	HMDB0001044	C00362
3-(3-Hydroxyphenyl)-3-hydroxypropanoic acid / 4-Hydroxyphenylla	HMDB0002643	
3-Hydroxybutyric acid	HMDB0000011	C01089
3-Hydroxyhexadecanoylcarnitine	HMDB0061642	
3-Hydroxyisobutyric acid	HMDB0000023	C06001
3-Hydroxyisovaleric acid	HMDB0000754	
3-Hydroxyoctadecenoylcarnitine	HMDB0013339	
3-Methoxytyramine	HMDB0000022	C05587
3-Methoxytyrosine	HMDB0001434	
3-Phosphoglyceric acid	HMDB0000807	C00597
3,5-/5,8-Tetradecadiencarnitine	HMDB0013331	
4-Guanidinobutanoic acid	HMDB0003464	C01035
5-Hydroxyindoleacetic acid	HMDB0000763	C05635
5-Thymidylic acid	HMDB0001227	C00364
5'-Methylthioadenosine	HMDB0001173	C00170
7-Methylguanine	HMDB0000897	C02242
9-/2-Hexadecenoylcarnitine	HMDB0013207	
Acetylcarnitine	HMDB0000201	C02571
Adenosine	HMDB0000050	C00212
Adenosine monophosphate	HMDB0000045	C00020
Adenosine monophosphate (AMP)	HMDB0000045	C00020
AICAR	HMDB0001517	C04677
Alanine	HMDB0000161	C00041
Allantoin	HMDB0000462	C01551
alpha-Aminobutyric acid (AABA)	HMDB0000452	C02356
Alpha-ketoisovaleric acid	HMDB0000019	C00141
Arabinose	HMDB0029942	
Arachidic acid	HMDB0002212	C06425
Arachidonic acid	HMDB0001043	C00219
Arachidonoylcarnitine	HMDB0006455	
Aspartic acid	HMDB0000191	C00049
Asymmetric dimethylarginine	HMDB0001539	C03626
beta-Alanine	HMDB0000056	C00099
beta-Cyanoalanine	HMDB0060245	C02512
beta-Glycerophosphoric acid	HMDB0002520	C02979
Betaine	HMDB0000043	C00719
Biliverdin	HMDB0001008	C00500
Butyrylcarnitine	HMDB0002013	C02862

Caffeic Acid-13C9 [M-H]-		
Caffeine-trimethyl-13C3 [M+H]+		
Campesterol	HMDB0002869	C01789
Carnitine	HMDB0000062	C00318
Cellobiose	HMDB0000055	C06422
Cellotriose		C06219
Cervonylcarnitine	HMDB0006510	
Citric acid	HMDB0000094	C00158
Creatine	HMDB0000064	C00300
Cyclic AMP	HMDB0000058	C00575
Cysteinylglycine	HMDB0000078	C01419
Cytosine	HMDB0000630	C00380
D-Glucuronic acid	HMDB0000127	C00191
D-Ribose	HMDB0000283	C00121
Dehydroascorbic acid (DHAA)	HMDB0001264	C00425
delta-Tocopherol	HMDB0002902	C14151
Deoxyuridine	HMDB0000012	C00526
Docosahexaenoic acid	HMDB0002183	C06429
Docosapentaenoic acid (22n-3)	HMDB0006528	C16513
Docosapentaenoic acid (22n-6)	HMDB0001976	
Docosapentaenoylcarnitine	HMDB0006321	
Docosatetraenoic acid	HMDB0002226	C16527
Docosatetraenoylcarnitine	HMDB0240758	
Eicosadienoylcarnitine	HMDB0240747	
Eicosapentaenoic acid	HMDB0001999	C06428
Eicoseneoylcarnitine	HMDB0240746	
Fructose 6-phosphate	HMDB0000124	C00085
Fumaric acid	HMDB0000134	C00122
g-Glutamylisoleucine	HMDB0011170	
g-Glutamylleucine	HMDB0011171	
g-Glutamylmethionine	HMDB0034367	
g-Glutamyltryptophan	HMDB0029160	
g-Glutamyltyrosine	HMDB0011741	
gamma-Aminobutyric acid	HMDB0000112	C00334
gamma-Aminobutyric acid (GABA)	HMDB0000112	C00334
gamma-Tocopherol	HMDB0001492	C02483
Gluconic acid	HMDB0000625	C00257
Glucose	HMDB0000122	C00221
Glucose 6-phosphate	HMDB0001401	C00092
Glutamic acid	HMDB0003339	C00217
Glutamine	HMDB0000641	C00064
Glyceric acid	HMDB0000139	C00258
Glycerol 3-phosphate	HMDB0000126	C00093
Glycyl-glycine	HMDB0011733	C02037
Guanine	HMDB0000132	C00242
Guanosine	HMDB0000133	C00387
Guanosine monophosphate	HMDB0001397	C00144

Hexanoylcarnitine	HMDB0000756	
Hexose	HMDB0000211	C00137
Homocitrulline	HMDB0000679	C02427
Hydroxyisovalerylcarnitine	HMDB0061189	
Hypoxanthine	HMDB0000157	C00262
Icosapentaenoylcarnitine	HMDB0241609	
Icosatrienoylcarnitine	HMDB0241578	
Indoleacetaldehyde	HMDB0001190	C00637
Indoxyl sulfate	HMDB0000682	
Inosine	HMDB0000195	C00294
Inosinic acid	HMDB0000175	C00130
Isobutyrylcarnitine	HMDB0000736	
Isoleucine	HMDB0000172	C00407
Isoleucyl-Isoleucine	HMDB0028910	
Isoleucyl-Leucine	HMDB0028911	
Isovalerylcarnitine	HMDB0000688	
Itaconic acid	HMDB0002092	C00490
Ketoleucine	HMDB0000695	C00233
L-Arginine	HMDB0000517	C00062
L-Aspartic acid	HMDB0000191	C00049
L-Glutamine	HMDB0000641	C00064
L-Isoleucine	HMDB0000172	C00407
L-Kynurenine	HMDB0000684	C00328
L-Leucine	HMDB0000687	C00123
L-Lysine	HMDB0000182	C00047
L-Methionine	HMDB0000696	C00073
L-Phenylalanine	HMDB0000159	C00079
L-Proline	HMDB0000162	C00148
L-Tryptophan	HMDB0000929	C00078
L-Tyrosine	HMDB0000158	C00082
L-Valine	HMDB0000883	C00183
Lactic acid	HMDB0000190	C00186
Leucine	HMDB0000687	C00123
Leucyl-Aspartate / g-Glutamylvaline	HMDB0028925	
Leucyl-Leucine	HMDB0028933	C11332
Linoleic acid	HMDB0000673	C01595
Linolenylcarnitine	HMDB0006318	
Linoleylcarnitine	HMDB0006469	
LysoPC(0:0/14:0)		C04230
LysoPC(0:0/15:0)		C04230
LysoPC(0:0/16:0)	HMDB0240262	C04230
LysoPC(0:0/16:1)		C04230
LysoPC(0:0/17:0)		C04230
LysoPC(0:0/18:0)	HMDB0011128	C04230
LysoPC(0:0/18:1)	HMDB0061701	C04230
LysoPC(0:0/18:2)	HMDB0061700	C04230
LysoPC(0:0/20:2)		C04230

LysoPC(0:0/20:3)		C04230
LysoPC(0:0/20:4)		C04230
LysoPC(0:0/20:5)		C04230
LysoPC(0:0/22:4)		C04230
LysoPC(0:0/22:5)		C04230
LysoPC(0:0/22:6)		C04230
LysoPC(14:0/0:0)	HMDB0010379	C04230
LysoPC(15:0/0:0)	HMDB0010381	C04230
LysoPC(16:0/0:0)	HMDB0010382	C04230
LysoPC(16:1/0:0)	HMDB0010383	C04230
LysoPC(17:0/0:0)	HMDB0012108	C04230
LysoPC(18:0/0:0)	HMDB0010384	C04230
LysoPC(18:1/0:0)	HMDB0002815	C04230
LysoPC(18:2/0:0)	HMDB0010386	C04230
LysoPC(18:3/0:0)	HMDB0010388	C04230
LysoPC(20:2/0:0)	HMDB0010392	C04230
LysoPC(20:3/0:0)	HMDB0010394	C04230
LysoPC(20:4/0:0)	HMDB0010395	C04230
LysoPC(20:5/0:0)	HMDB0010397	C04230
LysoPC(22:4/0:0)	HMDB0010401	C04230
LysoPC(22:5/0:0)	HMDB0010402	C04230
LysoPC(22:6/0:0)	HMDB0010404	C04230
LysoPC(O-16:0/0:0)		C04230
LysoPC(P-18:0/0:0)	HMDB0013122	C04230
LysoPE(0:0/16:0)	HMDB0011473	
LysoPE(0:0/18:1)	HMDB0011476	
LysoPE(0:0/18:2)	HMDB0011477	
LysoPE(0:0/20:4)	HMDB0011487	
LysoPE(0:0/20:5)	HMDB0011489	
LysoPE(0:0/22:4)	HMDB0011493	
LysoPE(0:0/22:5)	HMDB0011495	
LysoPE(0:0/22:6)	HMDB0011496	
LysoPE(16:0/0:0)	HMDB0011503	
LysoPE(18:0/0:0)	HMDB0011130	
LysoPE(18:1/0:0)	HMDB0011506	
LysoPE(18:2/0:0)	HMDB0011507	
LysoPE(20:1/0:0)	HMDB0011512	
LysoPE(20:4/0:0)	HMDB0011517	
LysoPE(20:5/0:0)	HMDB0011519	
LysoPE(22:5/0:0)	HMDB0011524	
LysoPE(22:6/0:0)	HMDB0011526	
LysoPE(P-16:0/0:0)	HMDB0011152	
LysoPE(P-18:0/0:0)	HMDB0240598	
Malic acid	HMDB0000156	C00149
Maltose	HMDB0000163	C00208
Maltotriose	HMDB0001262	C01835
Methionine	HMDB0000696	C00073

Methylbutanethioic acid	HMDB0061879	
myo-Inositol	HMDB0000211	C00137
Myo-Inositol 1-Monophosphate	HMDB0000213	C01177
Myristoleoylcarnitine	HMDB0240588	
Myristoylcarnitine	HMDB0005066	
N-(3-acetamidopropyl)pyrrolidin-2-one	HMDB0061384	
N-Acetyl-L-aspartic acid	HMDB0000812	C01042
N-Acetyl-L-methionine	HMDB0011745	C02712
N-Acetyl-L-phenylalanine	HMDB0000512	C03519
N-Acetyl-L-tyrosine	HMDB0000866	C01657
N-Acetylaspartylglutamic acid	HMDB0001067	C12270
N-Acetylneuraminic acid	HMDB0000230	C19910
N-Acetylserine	HMDB0002931	
Niacinamide	HMDB0001406	C00153
Octenoylcarnitine	HMDB0241701	
Oleic acid / Elaidic acid	HMDB0000207	C00712
Oleoylcarnitine	HMDB0005065	
Ophthalmic acid	HMDB0005765	
Ornithine	HMDB0000214	C00077
Oxoglutaric acid	HMDB0000208	C00026
Palmitoylcarnitine	HMDB0000222	C02990
Pantothenic acid	HMDB0000210	C00864
Phenylalanine	HMDB0000159	C00079
Phenylalanine-13C9 [M-H]-		
Phenylalanine-13C9 [M+H]+		
Phenylalanylalanine	HMDB0028988	
Phenylalanylglutamic acid / g-Glutamylphenylalanine	HMDB0028994	
Phenylalanylisoleucine	HMDB0028998	
Phenylalanylleucine	HMDB0013243	
Phenylalanylphenylalanine	HMDB0013302	
Phenylalanyltryptophan / Tryptophyl-Phenylalanine	HMDB0029006	
Phosphoenolpyruvic acid	HMDB0000263	C00074
Phosphoethanolamine	HMDB0000224	C00346
Proline	HMDB0000162	C00148
Prolylhydroxyproline / Pyroglutamylvaline	HMDB0006695	
Propionylcarnitine	HMDB0000824	C03017
Pterin	HMDB0000802	C00715
Pyroglutamic acid	HMDB0000267	C01879
Pyruvic acid	HMDB0000243	C00022
Ribose	HMDB0000283	C00121
Ribose 5-phosphate	HMDB0001548	C03736
S-Adenosylhomocysteine	HMDB0000939	C00021
Salicylic acid-D4 [M-H]-		
Serotonin	HMDB0000259	C00780
Spermidine	HMDB0001257	C00315
Spermine	HMDB0001256	C00750
Sphingosine 1-phosphate	HMDB0000277	C06124

Squalene	HMDB0000256	C00751
Stearoylcarnitine	HMDB0000848	
Succinyladenosine	HMDB0000912	
Taurochenodesoxycholic acid	HMDB0000951	C05465
Taurocholic acid	HMDB0000036	C05122
Threonic acid	HMDB0000943	C01620
Threonine	HMDB0000167	C00188
Tiglylcarnitine	HMDB0002366	
Tyrosine	HMDB0000158	C00082
Uracil	HMDB0000300	C00106
Uric acid	HMDB0000289	C00366
Uridine 5-monophosphate		
Uridine 5'-monophosphate	HMDB0000288	C00105
Uridine/Pseudouridine	HMDB0000296	C00299
Valerylcarnitine	HMDB0013128	
Valine	HMDB0000883	C00183
Xanthine	HMDB0000292	C00385
Xanthosine	HMDB0000299	C01762

REFERENCES

1. A J, Trygg J, Gullberg J, et al. Extraction and GC/MS analysis of the human blood plasma metabolome. *Anal Chem.* 2005;77(24):8086-8094.
2. Gullberg J, Jonsson P, Nordstrom A, Sjostrom M, Moritz T. Design of experiments: an efficient strategy to identify factors influencing extraction and derivatization of Arabidopsis thaliana samples in metabolomic studies with gas chromatography/mass spectrometry. *Anal Biochem.* 2004;331(2):283-295.
